# The landscape of chromosomal aberrations in breast cancer mouse models reveals driver-specific routes to tumorigenesis

**DOI:** 10.1038/ncomms12160

**Published:** 2016-07-04

**Authors:** Uri Ben-David, Gavin Ha, Prasidda Khadka, Xin Jin, Bang Wong, Lude Franke, Todd R. Golub

**Affiliations:** 1 Cancer Program, Broad Institute of Harvard and MIT, Cambridge, Massachusetts 02142, USA; 2 Department of Medical Oncology, Dana-Farber Cancer Institute, Boston, Massachusetts 02115, USA; 3 Department of Genetics, University of Groningen, Groningen 9711, The Netherlands; 4 Harvard Medical School, Harvard University, Boston, Massachusetts 02115, USA; 5 Howard Hughes Medical Institute, Chevy Chase, Maryland 208115, USA

## Abstract

Aneuploidy and copy-number alterations (CNAs) are a hallmark of human cancer. Although genetically engineered mouse models (GEMMs) are commonly used to model human cancer, their chromosomal landscapes remain underexplored. Here we use gene expression profiles to infer CNAs in 3,108 samples from 45 mouse models, providing the first comprehensive catalogue of chromosomal aberrations in cancer GEMMs. Mining this resource, we find that most chromosomal aberrations accumulate late during breast tumorigenesis, and observe marked differences in CNA prevalence between mouse mammary tumours initiated with distinct drivers. Some aberrations are recurrent and unique to specific GEMMs, suggesting distinct driver-dependent routes to tumorigenesis. Synteny-based comparison of mouse and human tumours narrows critical regions in CNAs, thereby identifying candidate driver genes. We experimentally validate that loss of Stratifin (*SFN*) promotes *HER2*-induced tumorigenesis in human cells. These results demonstrate the power of GEMM CNA analysis to inform the pathogenesis of human cancer.

## Abstract (web summary)

Genetically engineered mouse models of cancer are useful in identifying oncogenes and tumour suppressors. Here, the authors use gene expression profiles to generate a catalogue of copy number aberrations in 45 mouse models, and compare mouse and human tumours to identify additional drivers of tumorigenesis.

## Graphical abstract

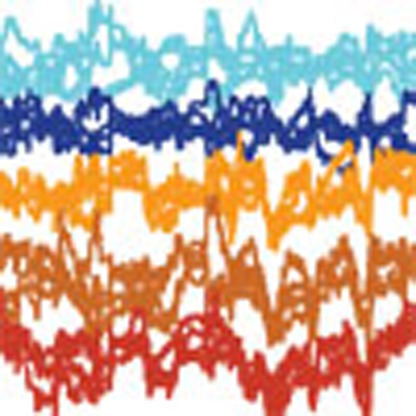

The understanding of cancer biology has benefitted tremendously from large-scale analyses of genomic data. Resources of comprehensive molecular characterizations of human tumours, best illustrated by The Cancer Genome Atlas (TCGA), have become indispensable for contemporary cancer research^1^. However, the utility of such data is limited by the extensive genetic diversity of the human population and by the complexity of late-stage tumours that harbour true driver events buried in a majority of passenger alterations. The study of aneuploidy and large copy-number alterations (CNAs), affecting on average ∼25% of the tumour genome^2^,^3^, is particularly challenging. As these CNAs often encompass hundreds of genes, it is difficult to distinguish driver from passenger genes within such aberrations. It is equally challenging to associate tumour-initiating events (for example, point mutations) with unique CNAs that cooperate with them on tumorigenesis.

In principle, genetically engineered mouse models (GEMMs) provide a strategy to overcome the limitations of human genomic data. GEMMs have been successfully used to dissect cellular and molecular aspects of tumorigenesis, to identify and validate candidate cancer genes, and to test new therapeutic approaches^4^,^5^. Mouse copy-number data at large scale could therefore facilitate the study of multiple aspects of tumour biology. However, the landscape of chromosomal aberrations in GEMMs has been underexplored, even in breast cancer, for which GEMMs have been generated and studied extensively^4^,^5^,^6^,^7^,^8^,^9^. We therefore set out to generate a comprehensive catalogue of chromosomal aberrations in breast cancer GEMMs, and to mine this resource to address multiple aspects of tumour development. We find that CNA prevalence, as well as the recurrence of specific events, are largely determined by the initiating perturbations. Building on this finding, we compare context-specific recurrent events between mouse models and human patients, and identify candidate co-driver genes. We experimentally validate the relevance of one such gene, Stratifin (*SFN*), to human *HER2*-induced tumorigenesis.

## Results

### Gene expression profiles reveal CNAs in breast cancer GEMMs

**Figure 1:**
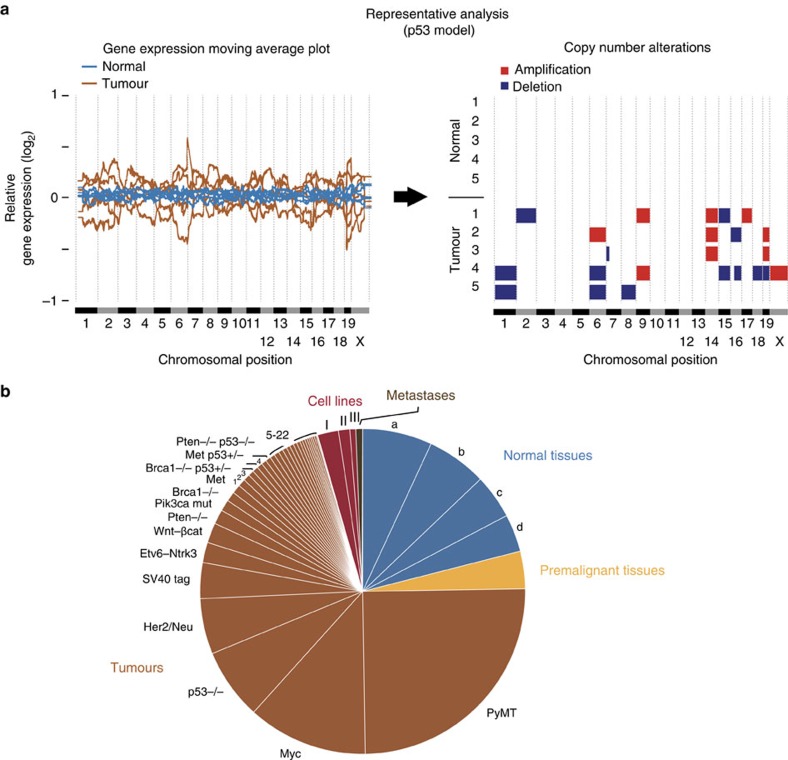
Analysing aneuploidy and large CNAs in breast cancer GEMMs using gene expression profiles. (**a**) A representative example of gene expression-based CNA analysis. Left: moving average plots of global gene expression levels along the genome of five normal mammary samples (blue lines) and five tumour samples (brown lines) from the p53^−/−^ model. Right panel: piecewise constant fit (PCF) detection of CNAs in the same samples based on coordinated deviations of gene expression levels throughout large genomic regions. Amplifications are shown in red, deletions in blue. (**b**) A pie chart describing the 2,697 gene expression profiles analysed: 567 normal tissue samples, 100 premalignant mammary tissues/lesions, 1,910 primary mammary tumours, 17 breast cancer metastases, and 103 breast cancer cell lines and cell line-derived tumours. These data were collected from 36 unique breast cancer mouse models. Letters represent normal tissues: a, non-mammary tissues from transgenic mice; b, mammary tissues from female control mice; c, non-mammary tissues from control mice; and d, mammary tissues from female transgenic mice. Roman letters represent cell lines and tumours derived from them: I, established breast cancer cell lines; II, cell line-derived tumours; and III, freshly derived cell lines. Tumours from GEMMs with >20 samples are presented by name, and numbers represent tumours from the remaining GEMMs: 1, Igf1r; 2, Apc^+/−^; 3, Pten^−/−^ p53^−/−^; 4, Hras; 5, Brg^+/−^; 6, Brca2^−/−^; 7, Stat5^−/−^; 8, Wnt Fgfi; 9, DMBA; 10, p53^+/−^ IR; 11, Rb^−/−^; 12, Int3/Notch4; 13, Brca1^+/−^ p53^+/−^ IR; 14, p18^−/−^; 15, LPA1; 16, Pten^−/−^ Her2/Neu; 17, Stat1^−/−^; 18, Atx; 19, Lpa2; 20, Lpa3; 21, Twist1 Kras; and 22, Pik3ca-mut p53^−/−^. See also Supplementary Fig. 1.

As copy-number data from breast cancer GEMMs are scarce, whereas genome-wide gene expression profiles from these GEMMs are abundant^10^,^11^,^12^, we first asked whether we could infer CNAs from their coordinated gene expression biases^13^,^14^,^15^,^16^,^17^. To examine this possibility, we modified the e-karyotyping method^15^ to analyse the mammary tumour gene expression data. For affymetrix microarray platforms, we also applied the functional genomic messenger RNA (mRNA) profiling (FGMP) method^17^. CNAs were estimated by analysing the differences in gene expression between normal mammary tissues and tumour samples (Methods). Analysis of 567 normal tissue samples led to 100% being accurately identified as diploid, suggesting a very low false detection rate of chromosomal aberrations (FDR<0.008, 95% CI). Furthermore, a comparison of expression-inferred CNAs to those estimated by comparative genomic hybridization (CGH) arrays from matched tumours, showed high concordance between these platforms: 26 out of 27 (96.3%) large (>5 Mb) CNAs identified by the RNA expression data were confirmed by DNA data (Supplementary Fig. 1). Therefore, gene expression-based analyses can capture the landscape of aneuploidy and large CNAs in tumours from breast cancer GEMMs, at approximately cytoband resolution (Fig. 1a).

Having validated the methodology, we were able to map the landscape of aneuploidy and large chromosomal aberrations in breast cancer GEMMs. For this aim, we collected and analysed gene expression profiles of 2,697 samples from 36 unique breast cancer mouse models: 567 normal tissue samples, 100 premalignant mammary tissues/lesions, 1,910 primary mammary tumours, 17 breast cancer metastases, and 103 breast cancer cell lines and cell line-derived tumours (Fig. 1b). These data were collected from 87 studies, across multiple experimental platforms, genetic backgrounds and transgene delivery methods, representing all major breast cancer GEMMs generated to date (Supplementary Data 1–6). The availability of this large-scale CNA resource allowed us to address a number of fundamental questions in cancer biology, as described below.

### CNAs arise late in breast cancer tumorigenesis

**Figure 2:**
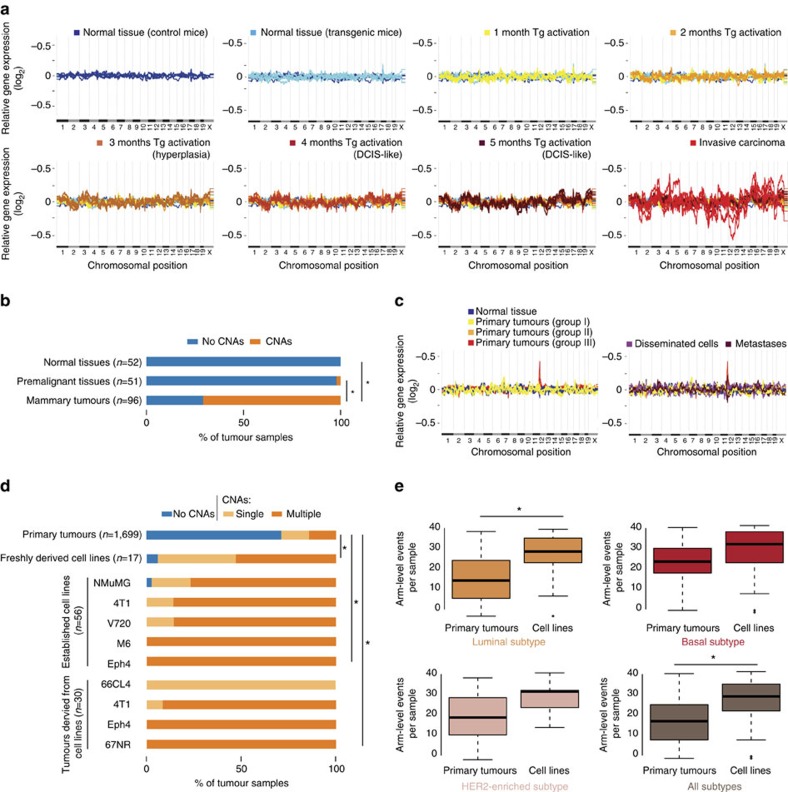
Chromosomal aberrations are a late event in breast cancer tumorigenesis and further aberrations are acquired during the derivation of cell lines. (**a**) Following chromosomal aberrations in the SV40Tag mouse model (GSE21444) reveals that large CNAs characterize the progression of non-malignant lesions to invasive carcinomas. Presented are moving average plots of gene expression profiles from various stages of tumour development. (**b**) Quantification of the prevalence of chromosomal aberrations in normal tissues (0/52), premalignant tissues (1/51) and invasive carcinomas (68/96) derived from the SV40Tag mouse model. *P=1.7 × 10^−15^ and *P<1 × 10^−16^ (*χ*^2^-test) for the comparison of tumours to normal and to premalignant tissues, respectively. (**c**) Following chromosomal aberrations in the PyMT mouse model (GSE43566) reveals that metastasis is not associated with an increased burden of aneuploidy and large CNAs. Presented are moving average plots of gene expression profiles from primary tumours (left; one aberration detected in 11 samples), and from disseminated cells and metastases (right; two aberrations detected in 22 samples). (**d**) Quantification of the prevalence of chromosomal aberrations in primary tumours (*n*=1,699), freshly derived cell lines (*n*=17), established cell lines (*n*=56) and cell line-derived tumours (*n*=30), revealing that cell lines exhibit an increased degree of chromosomal instability. *P=2 × 10^−10^, *P<1 × 10^−16^ and *P=2 × 10^−11^ (*χ*^2^-test) for the differences between primary tumours and freshly derived cell lines, established cell lines and cell line-derived tumours, respectively. Single, 1 CNA detected; multiple, >1 CNA detected. (**e**) Box plots presenting the number of arm-level CNAs in human primary breast tumours (from The Cancer Genome Atlas) and in human breast cancer cell lines (from the Cancer Cell Line Encyclopedia), divided by molecular subtype. *P=1.2 × 10^−4^ and *P=9.7 × 10^−7^ (Student's *t*-test) for the luminal subtype and for all subtypes combined, respectively. Boxes show the median, 25th and 75th percentiles, lower whiskers show data within 25th percentile −1.5 times the IQR, upper whiskers show data within 75th percentile +1.5 times the IQR and circles show outliers. See also Supplementary Figs 2–4.

We first explored the time course of CNA acquisition during breast cancer tumorigenesis in GEMMs. To address the fundamental, yet unanswered question of when CNAs arise, we analysed multiple studies for which data were available from distinct stages of mammary tumour development, including normal mammary tissues, premalignant lesions, ductal carcinomas *in situ* and invasive carcinomas. In SV40Tag-induced tumours, chromosomal aberrations were rarely detected in hyperplasias or ductal carcinomas *in situ*, but commonly found at the invasive carcinoma stage (Fig. 2a,b; Supplementary Fig. 2a). These findings suggested that chromosomal aberrations accumulated late during tumorigenesis. This observation was confirmed in five other GEMMs from which premalignant samples were available (Supplementary Fig. 2b; Supplementary Data 4). Therefore, aneuploidy and large CNAs are preferentially acquired, or become clonally dominant, during the progression of non-invasive lesions to invasive carcinomas, in line with recent findings from lung^18^ and skin^19^,^20^ cancer mouse models. In accordance, we observed a few instances, in which a genomic region did not meet our strict cutoff for CNA detection at early stages of tumorigenesis, but careful examination suggested that an aberration already existed at these time points in a subpopulation of cells, but was clonally selected only at the final stages of the tumour development (for example, loss of chromosome 2 in Supplementary Fig. 2).

We also examined aneuploidy and CNAs in metastases from Polyoma Middle T (PymT) and allografted tumours to determine whether additional chromosomal aberrations were required for the development of metastases (Supplementary Data 5). We did not detect an increased burden of chromosomal aberrations in these samples (Fig. 2c; Supplementary Fig. 2c), suggesting that further acquisition of such aberrations is not required for the metastatic phenotype.

### Cancer cell lines harbour more CNAs than primary tumours

As cancer cell lines are commonly used in the breast cancer research, it is important to assess the degree to which their genomic landscape faithfully represents that of primary tumours. Analysis of 103 samples from cell lines and cell line-derived tumours revealed that mouse breast cancer cell lines, as well as tumours generated following their transplantation, harbour many more CNAs compared with primary tumours (98% of the cell lines are aneuploid, compared with 29% of the tumours; Fig. 2d). Freshly derived cell lines are more than nine times more likely than their parental tumours to harbour chromosomal aberrations (Supplementary Fig. 2d), suggesting that cell line derivation is associated with the acquisition or selection of CNAs. Of note, distinct chromosomal aberrations are often detected in samples of the same established cell line (Supplementary Data 6), suggesting that additional chromosomal aberrations commonly arise during culture propagation. Similar to our findings in GEMMs, we found significantly more CNAs in human breast cancer cell lines, compared with human primary breast tumours of the respective subtype (Fig. 2e; Supplementary Fig. 3), in line with a previous finding with a much smaller data set^21^. Taken together, our analysis of breast cancer GEMMs reveals that the major wave of chromosomal aberrations occurs during the progression of a premalignant tissue to an invasive carcinoma; and that the prevalence of chromosomal aberrations in cell lines is much higher than in tumours (Supplementary Fig. 4). We therefore focused further analyses on the primary tumour samples.

### CNA prevalence is determined by the initial perturbation

**Figure 3:**
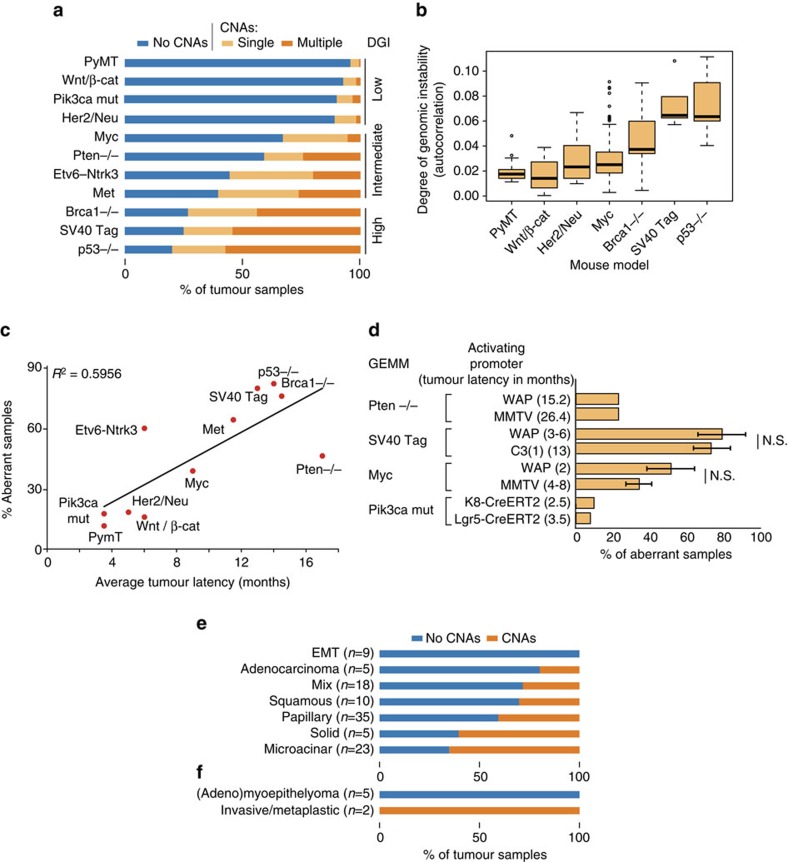
Driver-specific degree of genomic instability in breast cancer GEMMs. (**a**) The degree of genomic instability (DGI) differs considerably between breast cancer GEMMs. Presented is a quantification of CNA prevalence in the 11 most common GEMMs for which data were available from >20 samples from at least two independent studies. The Brca^−/−^, Pten^−/−^ and Met models represent both p53^+/+^ and p53^+/−^ backgrounds. Single, 1 CNA detected; multiple, >1 CNA detected. (**b**) The degree of genomic instability, as estimated by autocorrelation between proximate genes, in the Affymetrix mouse genome 430A 2.0 array. Note that the different DGI measures in **a** and in **b** result in the same GEMM ranking. Boxes show the median, 25th and 75th percentiles, lower whiskers show data within 25th percentile −1.5 times the IQR, upper whiskers show data within 75th percentile +1.5 times the IQR and circles show outliers. (**c**) A correlation between DGI and average tumour formation latency across GEMMs. Average tumour latencies depend on the activating promoters, and represent either MMTV (for Myc, PyMT, Her2/Neu, Wnt/βcat and Met), WAP (for SV40Tag and Etv6–Ntrk3) or Lgr5 (for Pik3ca_mut). (**d**) DGI is inherent to the driver gene, regardless of the promoter used for its activation/perturbation, and regardless of tumour latency within the GEMM. NS, not significant (Student's *t*-test). Bar plots represent the mean±s.d. (**e**) A significant difference in the DGI of histologically distinct tumours (GSE15904) induced by *Myc* in mice that share the same genetic background, when using the same promoter for *Myc* activation. (**f**) A significant difference in the DGI of histologically distinct tumours (GSE69290) induced by mutated *Pik3ca* in mice that share the same genetic background, when using the same promoter for mutated *Pik3ca* activation. See also Supplementary Figs 5 and 6.

A key question in cancer biology is whether particular initiating oncogenic events determine the eventual CNA landscape of the tumour. This question is particularly well suited to mouse models, where genetic background can be controlled, tumours can be generated by manipulating a single gene and the initiating event is known *a priori*. We therefore measured CNA prevalence in the 11 most common breast cancer GEMMs, and used it as an index of their degree of genomic instability, commonly referred to as DGI^17^. We found that DGI markedly differed between breast cancer GEMMs (*P*<10^−16^, *χ*^2^-test of independence), with the prevalence of large CNAs ranging from ∼4% to ∼80% of the tumours (>17-fold difference, for the PyMT and p53^−/−^ models, respectively; Fig. 3a). The three most unstable models (p53^−/−^, SV40Tag and Brca1^−/−^) often harboured multiple chromosomal aberrations per tumour. In contrast, the most stable models (PyMT, Wnt/βcat, Pik3ca_mut and Her2/Neu) primarily gave rise to diploid tumours, and almost never developed tumours with more than one CNA (Supplementary Fig. 5). The other four models (c-Myc, Pten^−/−^, Etv6–Ntrk3 and Met) showed intermediate prevalence of chromosomal aberrations. As expected, not only was p53^−/−^ the least stable model, but p53 status (^+/+^ or ^+/−^) was also a predictor of genomic instability across various models (Supplementary Fig. 6a). We assessed DGI in two additional ways: by computing ‘autocorrelation values' to determine the instability from correlated expression of neighbouring genes^17^, and by counting the number of CNA-encompassed genes (Methods). These analyses corroborated the significant DGI differences between the various mouse models (Fig. 3b; Supplementary Fig. 6b,c; Supplementary Table 1).

The availability of large-scale CNA data allowed us to revisit questions that had been previously addressed at smaller scale. For example, a recent study of 82 mice from three models of lung cancer reported a significantly higher level of aneuploidy and CNAs in GEMM tumours compared with chemically induced tumours, leading to the conclusion that genetically engineered and carcinogen-induced models develop tumours through different routes^18^. We re-addressed this question in 1,910 mouse mammary tumours from which the CNA data were inferred. In contrast to the reported result, we found that mammary tumours induced by the strong carcinogen 7,12-dimethylbenzanthracene fell well within the DGI spectrum of the genetic models. In fact, some genetic models exhibited even fewer chromosomal aberrations than the carcinogen-induced model (Supplementary Fig. 6d), arguing against a dichotomous distinction between chemical and genetic tumorigenesis routes.

Our large-scale data set also allowed us to dissect several variables that could affect driver-specific DGI. For example, as each GEMM has its typical tumour latency^22^, the DGI of each model might merely reflect the time it takes from transgene activation to tumour development. However, while DGI and average tumour latency correlated well (*R*^2^=0.6) across models (Fig. 3c), tumour latency had no effect on DGI within models (Fig. 3d). Similarly, DGI was not associated with mouse genetic background (Supplementary Fig. 6e,f), or with the method used for genetic perturbation (that is, the promoter used for transgene activation or excision; Fig. 3d). Therefore, DGI is intrinsic to the introduced perturbation, and is consistent within each model across genetic backgrounds, tumour latencies and activating promoters—an observation nearly impossible to make in human tumours given the diversity of genetic backgrounds and the diversity of inciting oncogenes. In contrast, we found DGI to be associated with tumour histological subtypes, in cases where the same transgene could give rise to histologically distinct tumours: a statistically significant DGI difference exists between subtypes of tumours induced by *Myc*^10^,^23^ (*P*=0.017, *χ*^2^-test of independence; Fig. 3e), and between subtypes of tumours induced by mutated *Pik3ca*^24^ (*P*=0.02, Fisher's exact test; Fig. 3f). We conclude that distinct tumour subtypes, generated by activating the same transgene, differ in their tendency to acquire CNAs.

### Specific drivers are associated with unique recurrent CNAs

**Figure 4:**
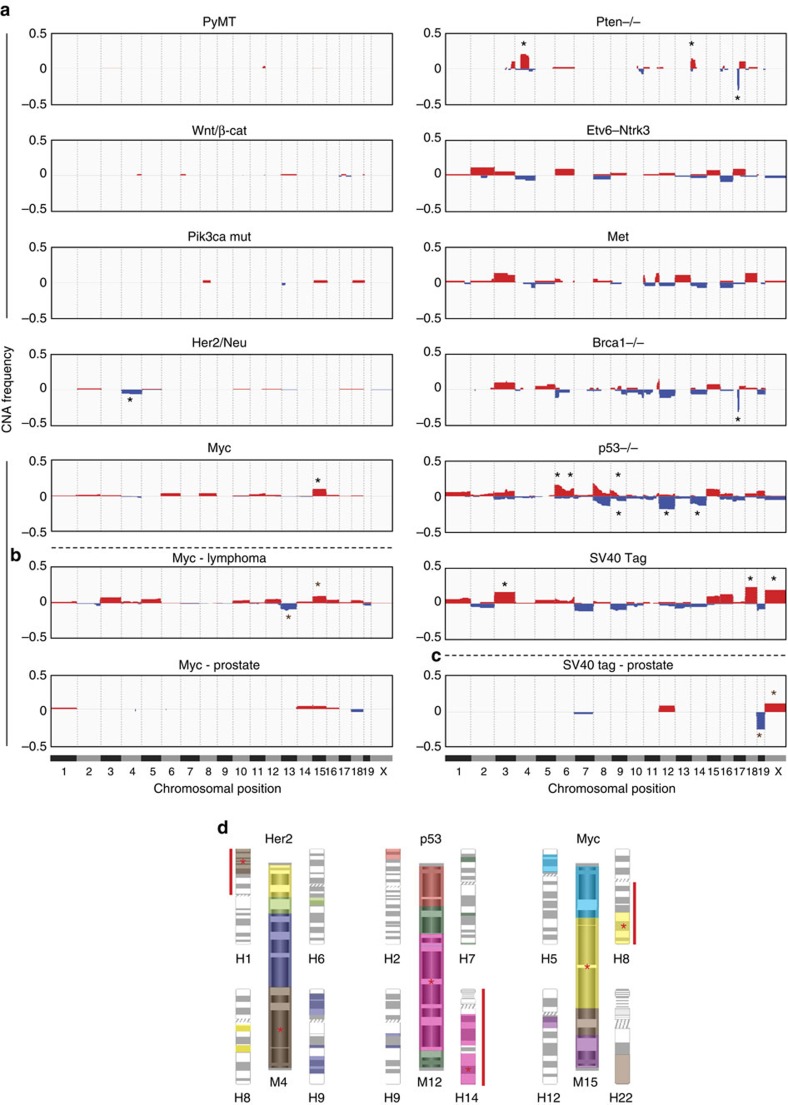
The landscapes of aneuploidy and large CNAs in breast cancer GEMMs reveal driver-specific recurrent events. (**a**) Frequency plots of chromosomal aberrations in each of the 11 GEMMs analysed, showing that each GEMM has a characteristic landscape of aneuploidy and large CNAs. Gains are shown in red, losses in blue. The 15 statistically significant driver-specific CNAs (adjusted *P*<0.05; *χ*^2^-test) are highlighted with black asterisks. (**b**) Frequency plots of chromosomal aberrations in lymphomas and in prostate tumours induced by *Myc* activation, showing that trisomy 15 recurs in *Myc*-induced tumours in various tissues, whereas other events (for example, monosomy 13 in lymphomas) are tissue dependent. Significant CNAs (adjusted *P*<0.05; *χ*^2^-test) are highlighted with brown asterisks. (**c**) Frequency plots of chromosomal aberrations in prostate tumours induced by SV40Tag, showing that trisomy X, and potentially monosomies 7 and 19, recur in SV40Tag-induced tumours independent of the tissue type, whereas other events (for example, trisomies 3 and 18 in breast tumours) are tissue dependent. Significant CNAs (adjusted *P*<0.05; *χ*^2^-test) are highlighted with brown asterisks. (**d**) Comparative oncogenomics can narrow regions of interest within recurrent CNAs in both species. Presented is a synteny analysis of three driver-specific CNAs: mouse chromosomes are shown in the centre, and syntenic human chromosomes surround them. Synteny blocks (>300 kb; small gaps filled) are color coded. Significantly, enriched CNAs in human tumours that activate the same pathway (as judged by gene expression signatures^33^) are marked with a red line to the side of the human chromosome. The synteny blocks that correspond to recurrent events in both species are marked with red asterisks. For example, trisomy 15 recurs in *Myc*-induced mouse breast cancer; as 8q amplification recurs in human tumours with high *MYC* expression signature, but only a telomere-bound part of 8q is syntenic to mouse chromosome 15, the region of interest within human chromosome 8q can be thus considerably narrowed (∼50% reduction in size). Of note, *MYC* itself is located within this syntenic region. See also Supplementary Figs 7–9.

We next asked whether the large-scale CNA data would enable us to identify statistically significant recurrent CNAs. Associating specific CNAs with specific tumour-initiating events could have far-reaching implications for understanding oncogenesis, with potential impact on targeted therapies. We therefore asked whether breast cancer GEMMs differ in their patterns of recurrent events. Indeed, we found distinct landscapes of chromosomal aberrations across models (Fig. 4a; Supplementary Fig. 7). To determine the recurrent CNAs in each model, we combined absolute and relative criteria: aberrations were determined as recurrent if present in at least 10% of the tumours, or if statistically significant in a binomial test (Bonferroni corrected *P*<0.05; Methods). Thirty five recurrent events were identified in the 11 common GEMMs, and 34 of them were confirmed to be significant by GISTIC2.0 analysis (Supplementary Fig. 8; Supplementary Table 2). To distinguish between cross-model and model-specific recurrent aberrations, we applied a *χ*^2^-test of independence, thus identifying 15 unique, GEMM-specific CNAs (Fig. 4a; Supplementary Table 2). These analyses revealed that each GEMM is associated with a characteristic chromosomal landscape, suggesting that these CNAs are not simply passenger events, but rather play a functional role in promoting tumorigenesis.

To examine whether recurrent driver-specific CNAs are also tissue dependent, we took advantage of common oncogenes that can induce cancer in multiple tissues. Specifically, we analysed 319 Myc-induced lymphoma and prostate tumours (Supplementary Data 7), as well as 92 SV40Tag-induced prostate tumours (Supplementary Data 8). With both transgenes, some recurrent CNAs were observed only in a particular cancer type, whereas others recurred across multiple cancers (Fig. 4a–c), suggesting that a subset of driver-specific CNAs cooperate with the initial driver independently of the targeted tissue. Interestingly, the lymphoma and prostate data also recapitulated our findings in breast cancer that aneuploidy occurs late during cancer progression, and that DGI is inherent to the driver gene (Supplementary Fig. 9).

### Cross-species analysis identifies candidate co-driver genes

As genes important for tumorigenesis are likely to reside within recurrent CNAs, we next asked whether integrated analysis of CNAs and gene expression could uncover such driver genes. To identify oncogenes or tumour suppressor genes that promote tumorigenesis across models, we compared recurrent events shared by multiple GEMMs. Amplification of 11qE1–E2 is a recurrent event in three of the GEMMs: PyMT, Met and Brca1^−/−^ (Fig. 4a). We therefore searched for genes that reside within this region and that are significantly overexpressed in each of these models^10^. The anti-apoptotic gene *Survivin* (*Birc5*) was the only overlapping gene between the three GEMMs (Supplementary Fig. 10a), suggesting its potential involvement in breast cancer tumorigenesis. Interestingly, we found that three additional GEMMs (SV40Tag, Myc and Her2/Neu) in which 11qE1–E2 amplification was not recurrent, also significantly overexpressed *Birc5* (ref. 10), suggesting that its expression may be dysregulated through multiple mechanisms.

In line with a driving role for BIRC5 in human mammary tumorigenesis, this gene is commonly amplified in human invasive ductal breast carcinomas^25^, in human invasive lobular breast carcinomas^26^ and in human breast cancer xenografts^27^ (Supplementary Fig. 10b). Moreover, we found high expression of BIRC5 to be associated with worse clinical outcome in human breast cancer patients (Supplementary Fig. 10c), consistent with previous analyses of much smaller cohorts^28^,^29^. Lastly, knockdown of BIRC5-induced apoptosis and/or reduced colony formation capacity of breast cancer cell lines of the basal^30^, HER2^31^ and luminal^32^ subtypes, further supporting its subtype-independent oncogenic role in breast cancer.

To identify genes that promote tumorigenesis in a particular genomic context, we performed an integrated cross-species analysis. Model-specific CNAs may be driven by genes that cooperate, or interfere, with the initial driver event, and may be important for human tumorigenesis in the same genetic context. We therefore sought to take advantage of the incomplete synteny between the mouse and human genomes to narrow critical regions of interest. We compared the recurrent aberrations identified in GEMMs to those that characterize human breast cancers with activation of the same pathway^33^ (Methods). This comparison identified several syntenic recurrent events, enabling a focus on substantially smaller regions within large CNAs in both species (Fig. 4d; Supplementary Table 3). For example, we identified monosomy 4 as a recurrent event in the Her2/Neu GEMM. Mouse chromosome 4 is syntenic to four human chromosomes; of these, only chromosome 1p is commonly deleted in human tumours with a *HER2* amplification gene expression signature^33^. This approach led to a considerable narrowing of the critical region of deletion (60 and 45% reduction for mouse and human chromosomes, respectively; Fig. 4d). Focusing on this syntenic region, we next compiled a list of orthologous genes that reside within it and are downregulated in *Her2*/*HER2*-induced tumours (Supplementary Data 9; Methods). These candidate genes, together with *HER2* itself, were then subjected to unbiased gene network analyses using the GeNets platform (Methods), which identified *SFN* as a strong candidate gene to cooperate with *HER2* during tumorigenesis (Supplementary Fig. 11a).

### Loss of *SFN* promotes human *HER2*-induced tumorigenesis

**Figure 5:**
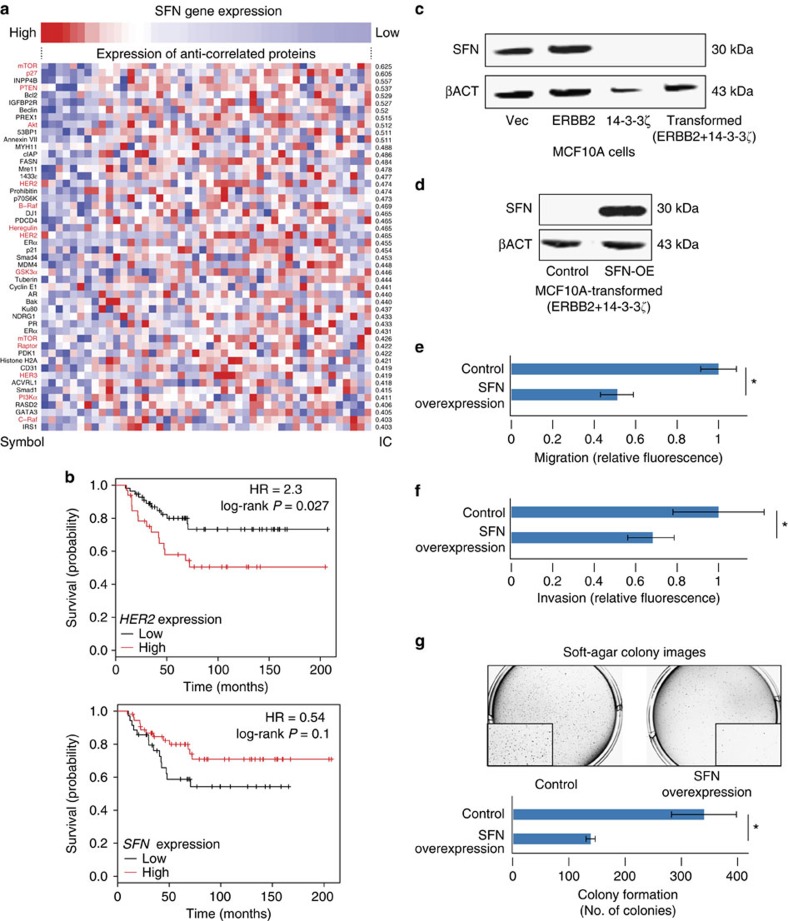
Downregulation of SFN promotes *HER2*-induced human breast cancer tumorigenesis. (**a**) *SFN* expression level is anti-correlated with the protein expression level of multiple members of the HER2 pathway in human breast cancer cell lines. Presented are the 50 most negatively associated proteins, the HER2 pathway members are labelled in red. IC, information coefficient. (**b**) Low expression of *SFN* and high expression of *HER2* are associated with worse prognosis in *HER2*-enriched subtype tumours. Presented are Kaplan–Meier plots of the patients' overall survival based on a limited cohort of 89 *HER2*-enriched subtype patients^46^. (**c**) Immunoblot analysis of SFN protein levels in stable MCF10A cell lines overexpressing an empty vector (MCF10A-Vec; non-tumorigenic), HER2 alone (MCF10A-ERBB2; non-tumorigenic), 14-3-3*ζ* alone (MCF10A-14-3-3*ζ*; non-tumorigenic) or both (MCF10A-ERBB2/14-3-3*ζ*; tumorigenic). Expression of 14-3-3*ζ* results in significant reduction of SFN expression. (**d**) Immunoblot analysis of SFN protein levels in transformed MCF10A cell lines. Overexpression of the *SFN* open reading frame restores SFN protein expression. (**e**) Decreased migration of transformed MCF10A cells following the restoration of SFN expression, as evaluated by a transwell migration assay. * *P*=1.5 × 10^−5^(Student's *t*-test). (**f**) Decreased invasion of transformed MCF10A cells following the restoration of SFN expression, as evaluated by a transwell invasion assay. *P=0.04 (Student's *t*-test). (**g**) Decreased colony formation of transformed MCF10A cells following the restoration of SFN expression, as evaluated by a soft-agar assay. Upper panel: images of colonies. Lower panel: quantification of the number of colonies in each condition. *P=0.04 (Student's *t*-test). Bar plots represent the mean±s.d. Experiments were performed in triplicates. See also Supplementary Figs 10–13.

*SFN* (*Stratifin*, also known as 14-3-3*σ*) has been described as a putative tumour suppressor involved in cell cycle progression and epithelial polarity^34^. However, in human breast cancer of the basal subtype, its expression has also been reported to promote invasiveness^35^, suggesting that its role in tumorigenesis (either oncogene or tumour suppressor) may be contingent on cellular context. To address this, we set out to determine whether deletion of SFN promotes or inhibits tumorigenesis in the human *HER2*-enriched breast cancer subtype. We found an inverse association between *SFN* mRNA expression levels and the protein levels of HER2, as well as that of multiple other proteins in the HER2 pathway, both in human breast tumours and in human breast cancer cell lines (Fig. 5a; Supplementary Fig. 11b). Furthermore, low SFN expression levels were associated with the decreased overall survival of breast cancer patients, specifically within the *HER2*-enriched human subtype (Fig. 5b; Supplementary Fig. 11c), most consistent with a loss-of-function, tumour suppressive role of SFN.

To functionally validate SFN's role in human *HER2*-induced tumorigenesis, we turned to a model of HER2-overexpressing human mammary epithelial cells. Overexpression of HER2 was not sufficient to transform MCF10A cells, unless combined with overexpression of 14-3-3*ζ*, another member of the 14-3-3 protein family^36^. Whereas control and HER2-overexpressing MCF10A cells expressed 14-3-3*σ*, its expression was lost upon overexpression of 14-3-3*ζ*, so that the transformed cells did not express it at all (Fig. 5c). Importantly, restoring SFN expression in the transformed cells significantly reduced their anchorage-independent colony formation capacity and their *in vitro* migration and invasion capabilities (Fig. 5d–g). Furthermore, we found that knockdown or knockout of SFN decreased the *in vitro* tumorigenicity of the basal subtype cell line MDA-MB-231, but had an opposite effect on two cell lines of the *HER2*-enriched subtype (MDA-MB-453 and EFM-192A) (Supplementary Fig. 12). Taken together, these results suggest that *SFN* acts as a tumour suppressor gene in the context of *HER2*-mediated transformation, in line with previous data from the mouse^37^ and in contrast to its role in non-*HER2*-driven human mammary tumours^35^. More broadly, these results delineate a comparative oncogenomics strategy to identify genes that co-drive tumorigenesis in specific genomic contexts (Supplementary Fig. 13). Applying the same strategy to the recurrent CNAs in additional mouse models yielded a list of candidate genes that may underlie each of these aberrations (Supplementary Data 9).

## Discussion

GEMMs make a powerful tool for *in vivo* modelling of human breast cancer. However, as GEMMs do not always recapitulate the progression of the human disease, comprehensive genomic characterizations of these models should inform their proper use in cancer research, and guide the selection of the most suitable GEMMs for addressing a particular biological question. Unlocking the copy-number information hidden in thousands of gene expression profiles allowed us to perform the first comprehensive study of aneuploidy and large CNAs in GEMMs. By systematically mining this novel resource (available as band-level aberration matrices in Supplementary Data 10), we uncovered a complex landscape of chromosomal aberrations in breast cancer GEMMs, indicative of driver-specific genomic routes to tumour development. We used this data set to address several long-standing questions in cancer research, and demonstrated its relevance to the human disease.

Several of our findings are of particular interest: First, we show that CNA prevalence varies extensively across mouse models, depending on the inciting oncogene. Westcott *et al.*^18^ recently concluded, based on the analysis of 82 tumours from three mouse lung cancer models, that there were systematic differences in CNA prevalence between genetically induced and chemically induced models of cancer. Our analysis of 1,910 mammary tumours, the largest ever reported, clearly shows that the variation across GEMMs is similar to that seen between some GEMMs and chemical models. It therefore emphasizes the importance of performing such analyses at the appropriate scale. Second, we demonstrate the feasibility of associating specific inciting oncogenic events with specific aneuploidies, even in mouse models that are otherwise genomically stable. These relationships can thus serve as a basis for discovering the multi-step pathogenesis of cancer. Third, we illustrate how cross-species CNA analyses can tease out driver genes within a large region of amplification or deletion in human tumours. Therefore, our findings demonstrate a novel approach to harness GEMM data to the understanding of human cancer pathogenesis.

Our findings reveal the context-dependent role of SFN (14-3-3*σ*) in human breast cancer. As 14-3-3 proteins interact with hundreds of binding partners and regulate multiple cellular processes, the molecular underpinnings of this unique behaviour remain to be elucidated. Previous studies showed that upregulation of transforming growth factor beta (TGFβ) is required for *HER2*-induced transformation of MCF10A cells^38^. Indeed, overexpression of 14-3-3*ζ* promotes *HER2*-induced tumorigenesis by activating the TGFβ pathway^36^. Here we report that overexpression of 14-3-3*ζ* also abolishes 14-3-3*σ* expression. Interestingly, 14-3-3*ζ* and 14-3-3*σ* play an opposite role in TGFβ-induced growth inhibition^39^, and 14-3-3*σ* was recently found to be a direct target of TGFβ^40^. This raises the intriguing possibility that loss of *SFN* promotes *HER2*-induced tumorigenesis through its modulation of the TGFβ pathway.

The same approach that identified *SFN* as a tumour suppressor in *HER2*-induced tumorigenesis was also applied to the systematic exploration of recurrent CNAs in other models, yielding a list of candidate genes that may underlie these driver-specific events. Interesting examples are the translation initiation factors and the ribosomal proteins that are co-amplified with *Myc* in both mouse and human *Myc*-induced tumours (Supplementary Data 9), which may collectively underlie the recurrence of 8q amplifications in human *MYC*-induced tumorigenesis; and PDZ binding kinase (*Pbk*), a gene previously shown to interact with p53 and modulate the expression of its transcriptional targets^41^, which is intriguingly deleted in both mouse and human p53-mutant tumours (Supplementary Data 9). The potential context-specific roles of these candidate genes await experimental validation. Importantly, as our approach for prioritizing candidate genes focuses on genes that interact with the driver event or belong to the same pathway (Supplementary Fig. 13), additional candidate genes may be identified by applying complementary approaches that focus on genes from alternative pathways.

In summary, our findings demonstrate the power of large-scale analyses of mouse models to inform the pathogenesis of mouse and human cancer. Further exploration of this resource, as well as its expansion to additional cancer types, should yield further insights into tumour biology.

## Methods

### Data assembly and processing

Gene expression profiles were obtained from the GEO (Gene Expression Omnibus) (http://www.ncbi.nlm.nih.gov/geo) and EMBL-EBI (European Molecular Biology Laboratory - European Bioinformatics Institute) (http://www.ebi.ac.uk) databases. Accession numbers are provided in Supplementary Data 1. Normalized matrix files were downloaded, and samples were curated manually according to the information available for each of them to identify the tissue type (normal, premalignant, primary tumour, metastasis, cell line or cell line-derived tumour), the tumour-initiating event, the promoter used for transgene activation or perturbation, and the mouse background strain. Arrays were analysed for quality control and the outliers were removed. The final database consisted of 567 normal tissue samples, 100 premalignant lesions, 1,910 invasive carcinomas, 103 cell lines and cell line-derived tumours, and 17 metastases from breast cancer GEMMs, as well as 319 samples and 92 samples from lymphoma and prostate GEMMs, respectively. GEMMs were defined according to the introduced/perturbed gene. The analysis was performed in batches, and normal tissue samples included in each study served as internal controls, whenever available. Data was processed using the R statistical software (http://www.r-project.org/)^42^: probe sets were organized by their chromosomal location, and the expression values were log2 transformed, if needed. Probe sets without annotated chromosomal location were removed. For genes with multiple probe sets, all the probe sets of the gene were averaged (as well as the chromosomal location) to obtain one intensity value per gene. Next, a threshold expression value was set, and genes with lower expression values were collectively raised to that level: flooring values were 6.5–7 for the Affymetrix and Illumina platforms, and −0.5 for the Agilent platforms. Probe sets not expressed in >20% of the samples within a batch were removed. The 10% of the probe sets with the most variable expression levels were also excluded, to reduce expression noise. Normalized CGH array data were also downloaded from the GEO website, and probe sets were organized by their chromosomal location.

### Inference of copy-number alterations

To infer CNAs from coordinated gene expression biases, the protocol developed by Ben-David *et al.*^15^ was applied. In each batch of analysis, the median expression of each gene across all normal tissue samples, or across the entire batch (if normal tissue samples were not available), was subtracted from the expression value of that gene in each sample to obtain a comparative value. The data were processed using a CGH analysis software program, CGH-Explorer (http://heim.ifi.uio.no/bioinf/Projects/CGHExplorer/)^43^. Gene expression regional biases were detected using the program's piecewise constant fit (PCF) algorithm, with the following set of parameters: least allowed deviation=0.15–0.4; least allowed aberration size=50–80; winsorize at quantile=0.001; penalty=12–18; and threshold=0.01. Moving average plots were generated with the moving average fit tool, with a window size of 200 genes. The DGI was subsequently determined for each sample based on the PCF results: either by counting the number of discrete aberrations within each sample (CNA prevalence-based DGI), or by counting the number of altered genes within each sample and dividing it by the total number of genes (gene-based DGI).

### Functional genomic mRNA profiling

For Affymetrix microarray platforms, mouse genome 430A, 430A 2.0 and 430 2.0 (which correspond to 53 of the 83 studies analysed), the FGMP method, proposed by Fehrmann *et al.*^17^ was also used. This procedure first estimates a set of transcriptional components that explain the majority of gene expression variation using a set of non-cancer samples. Upon correcting the gene expression data of cancer samples for these transcriptional components, the residual gene expression data strongly correlates with the copy number. We applied this approach to the mouse data, and corrected the mouse gene expression data for the first 25 principal components (PCs) that had been identified in a heterogeneous set of 17,081 mouse samples. The corrected data was then subjected to the same processing steps and CGH-PCF analysis described above to detect CNAs. The DGI was subsequently determined for each sample, by first sorting the 19,115 probe sets present in each of the three analysed Affymetrix platforms according to their genomic position, and then calculating (using a lag of 10 probe sets) the autocorrelation per sample, as described in Fehrmann *et al.*^17^

### Frequency plots and heat maps

CNAs were visualized using the Integrative Genomics Viewer (https://www.broadinstitute.org/igv/). The lists of segmented CNAs of all studies within each GEMM (received as outputs from the CGH-Explorer analyses) were united, and chromosomal locations were modified to match the mouse mm8 assembly. These lists were then uploaded to Integrative Genomics Viewer to generate frequency plots and heat maps.

### Recurrence analysis

To detect recurrent CNAs, the lists of segmented CNAs (CGH-Explorer output) for all studies within each GEMM were united and matched to the mouse chromosomal cytobands obtained from Ensembl 67 Archive for Mus musculus mm9 (May 2012). Each cytoband was assigned with the copy number of the segment(s) that correspond(s) to it (−1, deletion; 0, neutral; and 1, gain). The frequency of gains and losses of each chromosomal cytoband was computed within each GEMM. Aberrations were determined as recurrent if their prevalence was >10% in the *N* tumour samples, or if statistically significant (Bonferroni adjusted *P*<0.05) in binomial test for observing an alteration frequency *K*_c_ that is higher than expected. For the binomial test, the expected probability *p*_c_ of an event was computed as the background event frequency across all other cytobands within the GEMM, excluding the cytoband in the test: 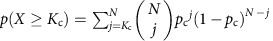. The test was performed separately for gains and for losses. To further improve our confidence in detecting recurrent CNAs, we applied GISTIC2.0 (version 2.0.22) using the Mus musculus (mm9) refSeq gene annotations (ftp://ftp.broadinstitute.org/pub/GISTIC2.0/refgenes/mm9_v0.2_refgene.tgz). As input segments already contained CNA calls (−1, deletion; 0, neutral; and 1, gain), the deletion and amplification thresholds were set at ±0.5, respectively. Other GISTIC parameters were the following: genegistic=1, maxseg=2,000, js=2, cap=1.5, broad=1, brlen=0.7, conf=0.99, armpeel=1, rx=0 and gcm=extreme. The *q* value of each cytoband was determined by the significant focal analysis (*q*<0.05), and if there were no significant focal overlaps, by the significant broad analysis (*q*<0.05). To determine model-specific recurrent CNAs, the Pearson's *χ*^2^-test of independence was applied, and aberrations were determined as model-specific if statistically significant (Bonferroni adjusted *P*<0.05) in this test.

### Detection of arm-level CNAs in human tumours and cell lines

The prevalence of aneuploidy and large CNAs was compared between 1,097 human breast cancer tumours from the TCGA project^25^ and 57 human breast cancer cell lines from the Cancer Cell Line Encyclopaedia (CCLE) cohort^44^, using GISTIC2.0 analysis of arm-level events^45^. Normalized, segmented Affymetrix single-nucleotide polymorphism 6.0 copy-number data for the cell lines were obtained from the CCLE (http://www.broadinstitute.org/ccle/data/browseData, 2012-04-05 hg18 dataset). Normalized, segmented single-nucleotide polymorphism 6.0 copy-number data for the TCGA breast adenocarcinoma samples were obtained from the TCGA/GDAC Firehose stddata__2014_10_17 data set (http://gdac.broadinstitute.org/runs/stddata__2014_10_17/data, doi:10.7908/C1K64H78). The data were median-centered and converted from log2 ratio to relative copy number by GISTIC2.0 (with cap=1.5). The median relative copy number across each chromosome arm was computed for every sample and compared with a threshold of ± 0.1 copies. Using the standard GISTIC2.0 noise threshold, arm median values exceeding 0.1 were assigned 1 in the output table, arm median values below −0.1 were assigned −1 and arm median values within the range were assigned 0.

### Synteny–orthology and gene networks analyses

Recurrent driver-specific aberrations identified in each GEMM were compared with recurrent aberrations identified in human breast tumours with high expression score of the same pathway^33^. Synteny between the mouse and human genome was determined and drawn using the synteny location-based display of the Ensemble Genome Browser (release 80) (http://www.ensembl.org). Genes that were significantly differentially regulated in the GEMM, compared with the normal tissue samples or compared with all other GEMMs^10^, were then filtered to include only the ones that have human orthologues that reside within the respective human syntenic region. Orthology between the mouse and human genes was examined using the HUGO (the Human Genome Organisation) Gene Nomenclature Committee comparison of orthology predictions search (http://www.genenames.org/cgi-bin/hcop). This list was then subjected to a gene network analysis, using GeNets: The Broad Institute Web Platform for Genome Networks (https://www.broadinstitute.org/genets). For each analysis, the original initiating gene of the model (for example, HER2, MYC, TP53 and so on) was added to the list of orthologous genes dysregulated within the recurrent CNAs of that particular model, and these gene lists were then subjected to a protein–protein interaction analysis using the InWeb3 network, and to a pathway analysis using the ConsensusPathDB network.

### Survival analysis

Survival data were obtained from the Kaplan–Meier Plotter breast cancer survival analysis database^46^, 2014 version (http://kmplot.com/analysis/index.php?p=service&cancer=breast). The mean expression values of the two ERBB2 probe sets (210930_s_at, 216836_s_at), the three SFN probe sets (33322_i_at, 33322_r_at, 209260_at) and the three BIRC5 probe sets (202094_at, 202095_s_at, 210334_x_at) were used. The *P* value was calculated using a log-rank test.

### Tumour latency analysis

Average tumour formation latencies were derived from The Jackson Laboratory website (http://jaxmice.jax.org/cancer/featured.html), and were confirmed by a recent review paper comparing latencies between different GEMMs^22^. The latencies characteristic of the Pten^−/−^, the Brca1^−/−^ and the Etv6–Ntrk3 GEMMs were derived from Liu *et al.*^47^, Li *et al.*^48^ and Diaz-Cruz *et al.*^49^, respectively.

### Association analysis of gene expression and protein levels

To assess the degree of association between mRNA expression levels of relevant genes, for example, *SFN*, and RPPA protein levels in cell lines and tumours we used an information-theoretic measure of association: the information coefficient. This quantity is a rescaling of the differential mutual information to make it lie in the interval [−1, 1] in a way similar to a correlation coefficient. The differential mutual information is a sensitive metric to detect linear and non-linear relationships between variables. The information coefficient, the matching score shown on the side of Fig. 5a; Supplementary Fig. 9c, is computed using standard kernel estimation procedures and its statistical significance (that is, nominal *P* values and false discovery rates) is assessed using an empirical permutation test. Similar association analysis has been applied in other problems, such as correlating drug sensitivities, mRNA levels, pathway profiles and genomic alterations^50^,^51^.

### Cell culture and genetic manipulations

MCF10A stable cell lines overexpressing an empty vector (control), ERBB2 alone, 14-3-3*ζ* alone or both (transformed MCF10A cells) were a kind gift from Dihua Yu and colleagues^36^. Cell lines were tested for mycoplasma contamination, and their morphology and performance in a functional (colony formation) assay were confirmed. MCF10A cells were cultured in MEGM Mammary Epithelial Cell Growth Medium (Lonza CC-3151), supplemented with the MEGM Bulletkit (Lonza CC-3151) and with 5 μg ml^−1^ human transferrin (Lonza CC-4205). MDA-MB-231, MDA-MB-453 and EFM-192A breast cancer cell lines were obtained from the Broad Institute CCLE repository^44^, and cultured in RPMI medium 1640 GlutaMAX (Thermo Fisher Scientific 61870-036). Lentiviral vector and its packaging vectors were transfected into 293T cells using FuGENE HD transfection reagent (Promega E2311). 293T cells were split into 6-cm^2^ plates, and were transfected the following day with 1 μg of vector, together with 100 ng of pCMV-VSV-G and 900 ng of psPAX2 packaging plasmids. For overexpression of SFN, the introduced vector was the CCSB-Broad Lentiviral Expression clone of Human SFN ORF (ccsbBroad304_06302; ccsbBroad304_99991 luciferase clone was used as control). For short hairpin RNA (shRNA)-mediated knockdown of SFN, the introduced vector was the GeneCopoeia HSH007802-LVRH1H shRNA-1; CSHCTR001-LVRH1H was used as a scrambled control shRNA. For CRISPR/Cas9-mediated knockout of SFN, the introduced vectors were the lentiCas9_blast and the lentiGuide_Puro into which a guide RNA (gRNA) against SFN was cloned; a gRNA against green fluorescent protein was used as control. The morning following transfection, the medium was replaced with fresh culture medium. Forty-eight and 72 h later, the lentivirus containing media was collected from transfection, filtered through a 0.45-μm filtre and the target cells were infected with the fresh lentivirus containing media (supplemented with 8 μg ml^−1^ polybrene). The next day, the medium was replaced with fresh culture medium containing selection antibiotics. MCF10A stable clones were selected with 5–10 μg ml^−1^ blasticidin (Life Technologies A11139-03); MDA-MB-231, MDA-MB-453 and EFM-192A stable clones were selected with 100–200 μg ml^−1^ hygromycin (for shRNAs; Life Technologies 10687-010), 1 μg ml^−1^ puromycin (for Cas9; Life Technologies A1113803) or 5–10 μg ml^−1^ blastocidin (for gRNAs; Life Technologies A11139-03). The sequences of the shRNAs are the following: shRNA-scrambled: GCTTCGCGCCGTAGTCTTA and shRNA-SFN: GCGAAACCTGCTCTCAGTA. The sequences of the gRNAs are the following: gRNA-green fluorescent protein: GGGCGAGGAGCTGTTCACCG and gRNA-SFN: CGAGATCGCCAACAGCCCCG.

### Immunoblotting

Total cell lysates were collected with a mix of 4 × protein loading buffer (Li-Cor 928-40004) and 10 × NuPAGE sample reducing agent (Life Technologies NP0009). The lysate was boiled for 5 min at 96 °C and frozen at −20 °C. Protein concentration was normalized between samples by cell counting. Cell lysates were subjected to electrophoresis using SDS–polyacrylamide gel electrophoresis and transferred to a nitrocellulose membrane with the iBlot2 dry blotting system (Life Technologies IB23001). Membrane was then blocked with Odyssey blocking buffer (Li-Cor 927-40100) for 1 h at room temperature, followed by an overnight primary antibody incubation at 4 °C in Odyssey blocking buffer with 0.1% Tween-20. For detection of SFN/14-3-3*σ*, we used the anti-human 14-3-3*σ* (E-11) mouse monoclonal antibody (Santa Cruz Biotechnologies, sc-166473, 1:200). For detection of β-actin, we used the anti-human β-actin rabbit polyclonal antibody (Santa Cruz Biotechnologies, sc-130656, 1:200). Following primary antibody staining, membranes were washed three times with Tris-Buffered Saline with Tween 20 (TBST) and incubated with the appropriate IRDye secondary antibody (Li-Cor) for 1 h at room temperature in Odyssey blocking buffer with 0.1% Tween-20 and 0.02% SDS. Membrane was then washed three times with TBST and twice with phosphate-buffered saline, and the signal was detected with a Li-Cor Odyssey CLx imaging machine and quantitated with the Image Studio software. Three biological replicates of the experiments were performed. Uncropped scans are presented in Supplementary Fig. 14.

### Soft-agar colony formation assay

Cells were suspended in 0.35% agar with their culture media, plated into six-well plates pre-coated with 0.5% agar at a density of 25 k cells per well and incubated at 37 °C. Once a week, 200 μl of media was added to each well. At 2 weeks, cells were stained for 1 h with 0.005% crystal violet (Sigma-Aldrich V5265) in phosphate-buffered saline with 4% formaldehyde, washed three times and images of the entire wells were taken using a Leica automated microscope with an ACE light source (Schott A20500). Images were analysed and colonies (>10 pixel units in size) were automatically counted using the Cell Profiler imaging software. Three biological replicates of the experiments were performed.

### Cell migration and invasion assays

CytoSelect 96-well cell migration assay (Cell Biolabs CBA-106) and CytoSelect 96-well cell invasion assay (Cell Biolabs CBA-112-COL) were performed according to the manufacturer's protocol. In short, cells were suspended in low-serum (0.5% fetal bovine serum) DMEM medium, and added to the top chambers of the 96-well cell migration plates or the collagen-coated 96-well cell invasion plates at a density of 50 k cells per well. Complete media was added to the bottom chambers as attractant. Twenty-four hours after incubation, migrating/invading cells were detached from the underside of the membrane using cell detachment solution, lysed with lysis buffer and stained with CyQuant GR dye solution. Fluorescence intensity was determined with Envision plate reader at 485/535 nm. Five biological replicates of the experiments were performed.

### Statistical analyses

The significance of the differences in the prevalence of CNAs between different stages of tumorigenesis, between primary tumours and cell lines, between the various GEMMs, between activating promoters, between genetic backgrounds, between histological subtypes, between tumours with different p53 status and between mouse strains was determined using the Pearson's *χ*^2^-test of independence, or using the Fisher's exact test (whenever the number of samples for one of the conditions was <10). The significance of the difference in the average number of arm-level CNAs between human primary tumours and cell lines, and the significance of the difference in the performance in the migration, invasion and colony formation assays were determined using the two-tailed Student's *t*-test. The significance of the difference between the autocorrelation distributions of GEMMs was determined by a Mann–Whitney *U* test. The significance of the difference between the DGI of the various GEMMs, as determined by the fraction of altered genes or by the number of discrete CNAs was determined by a Kruskal–Wallis rank-sum test, followed by a *post hoc* Dunn's test of multiple comparisons. Bar plots represent the mean ± s.d. Box plots were generated using the R statistical software, so that the boxes show the median, 25th and 75th percentiles, lower whiskers show data within 25th percentile −1.5 times the interquartile range (IQR), upper whiskers show data within 75th percentile +1.5 times the IQR and circles show outliers.

### Data availability

Data referenced in this study and their associated accession codes are available in Supplementary Data 1. The authors declare that any other data supporting the findings of this study are available within the article, its Supplementary Information files or available from the author upon request.

## Additional information

**How to cite this article:** Ben-David, U. *et al.* The landscape of chromosomal aberrations in breast cancer mouse models reveals driver-specific routes to tumorigenesis. *Nat. Commun.* 7:12160 doi: 10.1038/ncomms12160 (2016).

## Supplementary Material

Supplementary Information Supplementary Figures 1-14 and Supplementary Tables 1-3.

Supplementary Data 1 Breast cancer datasets analyzed in the current study.

Supplementary Data 2 List of breast cancer tumor samples.

Supplementary Data 3 List of normal mammary tissue samples.

Supplementary Data 4 List of premalignant mammary samples.

Supplementary Data 5 List of breast cancer metastase.

Supplementary Data 6 List of breast cancer cell lines.

Supplementary Data 7 Myc-induced tumors in lymphoma and prostate cancer GEMMs.

Supplementary Data 8 8: SV40Tag-induced tumors in prostate cancer GEMMs.

Supplementary Data 9 Candidate genes underlying recurrent driver-specific CNAs.

Supplementary Data 10 Band-level aberration matrix files.
