# Supplementary material for: The landscape of chromosomal aberrations in breast cancer mouse models reveals driver-specific routes to tumorigenesis
Source: Nat Commun. 2016 Jul 4;7:12160. doi: 10.1038/ncomms12160 (PMC4932194; doi:10.1038/ncomms12160)
Supplement: Supplementary Information — Supplementary Figures 1-14 and Supplementary Tables 1-3. [file ncomms12160-s1.pdf]

Supplementary Figure 1

a

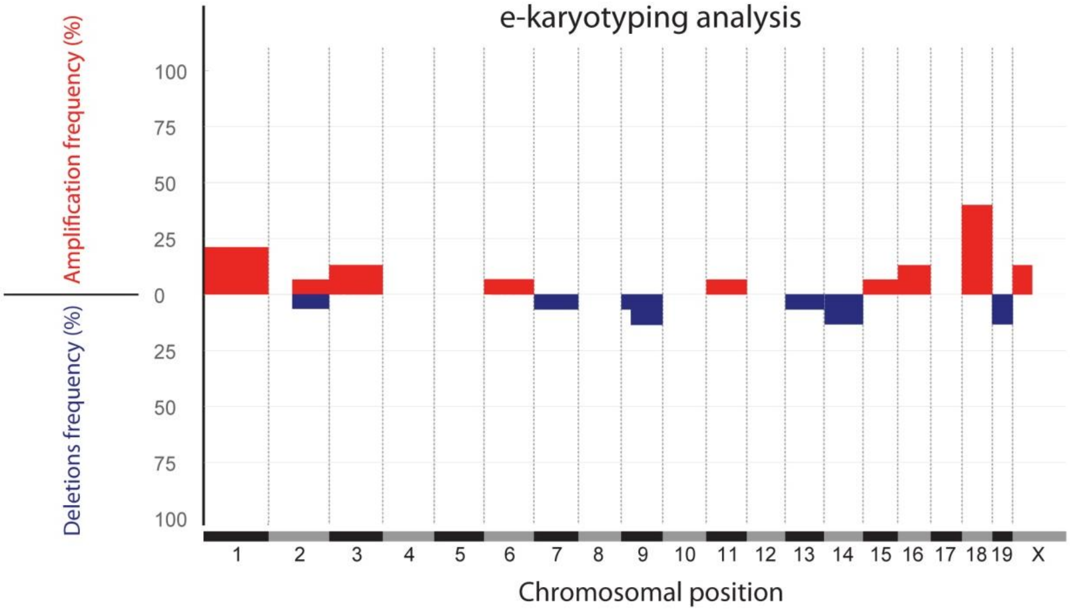

b

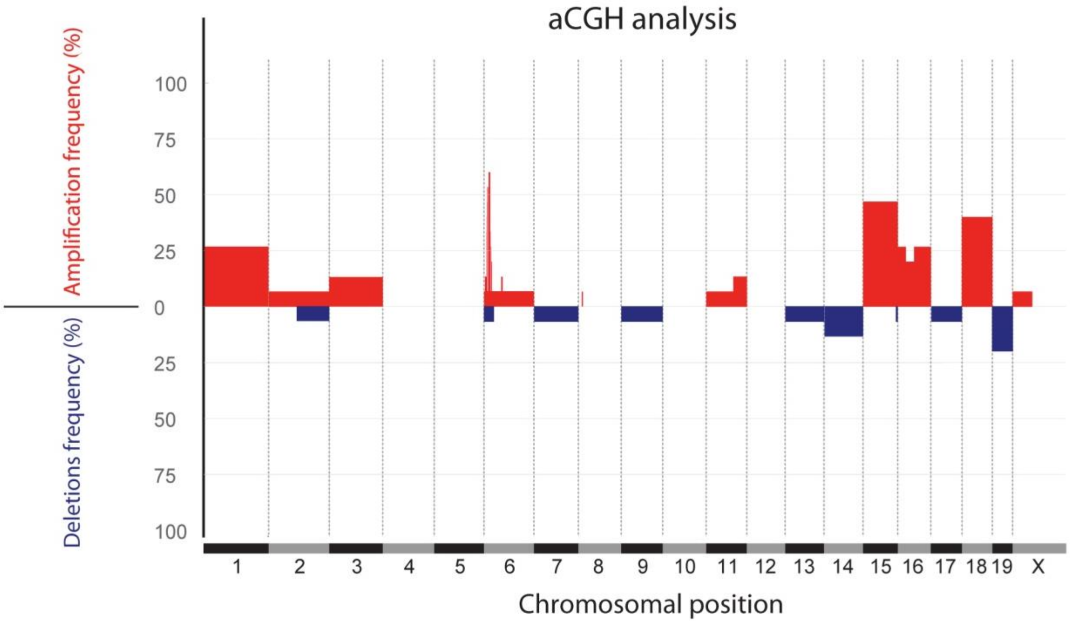

Supplementary Figure 1: Comparison of aCGH-based and expression-based CNA analysis of tumors from breast cancer GEMMs.

(**a**) CNA analysis of expression microarray data obtained from 15 tumors in the SV40Tag GEMM[52]. (**b**) CNA analysis of aCGA data obtained from the same tumors. 26 of 27 aberrations detected by expression analysis were also identified by the aCGH analysis (false detection rate of 0.037), demonstrating that expression-based karyotyping can accurately capture the landscape of aneuploidy and large CNAs in breast cancer GEMMs.

Supplementary Figure 2

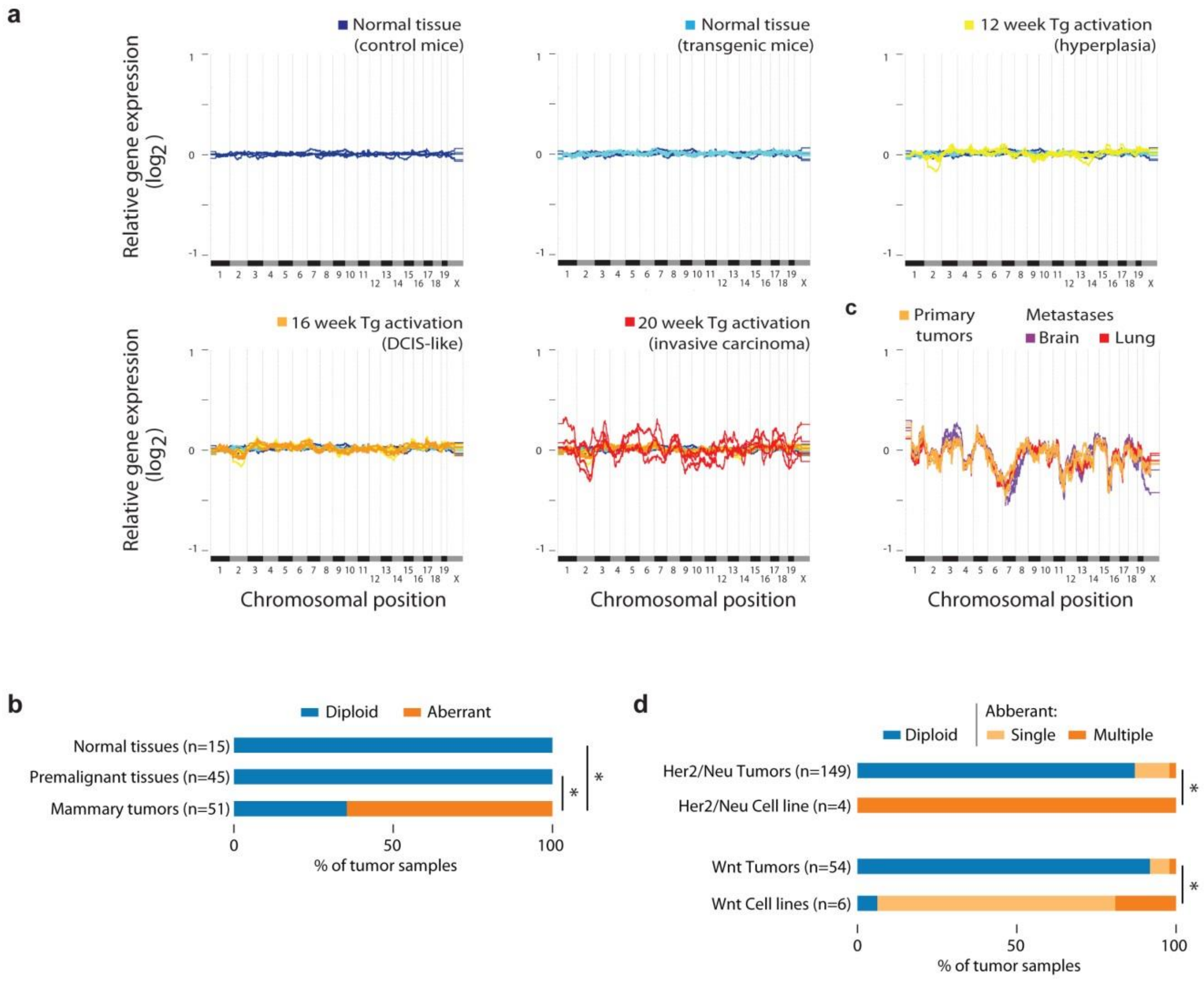

Supplementary Figure 2: Chromosomal aberrations are a late event in breast cancer tumorigenesis, and further aberrations are acquired during the derivation and propagation of cell lines.

(**a**) Following chromosomal aberrations in another study with the SV40Tag mouse model (GSE50813) confirms that large CNAs occur, or are selected, only at the progression of non-malignant lesions to invasive carcinomas. Presented are moving average plots of gene expression profiles from various stages of tumor development. (**b**) Quantification of the prevalence of chromosomal aberrations in normal tissues, premalignant tissues/lesions and invasive carcinomas derived from all mouse models combined (**Supplementary Data 4**; only samples from studies with CNA-harboring tumors are included). *, $p=1x10^{-5}$ and $p=2.7x10^{-11}$ (chi-squared test), for the comparison of tumors to normal and to premalignant tissues, respectively. (**c**) Following chromosomal aberrations in tumors derived by injecting 4T1 cells into recipient mice (GSE54773) confirms that metastasis is not necessarily associated with increased burden of aneuploidy and large CNAs. Presented are moving average plots of gene expression profiles from primary tumors and metastases. Only one additional CNA was detected in one out of six brain and lung metastases. (**d**) Freshly-derived cell lines harbor many more CNAs than the primary tumors from which they are derived. Quantification of the prevalence of chromosomal aberrations in primary tumors and in the cell lines derived from them, in two genomically stable GEMMs, Her2/Neu and Wnt/βcat. *, $p=2x10^{-4}$ and $p<1x10^{-16}$ (Fisher's exact test) for Her2/Neu and Wnt/βcat, respectively.

Supplementary Figure 3

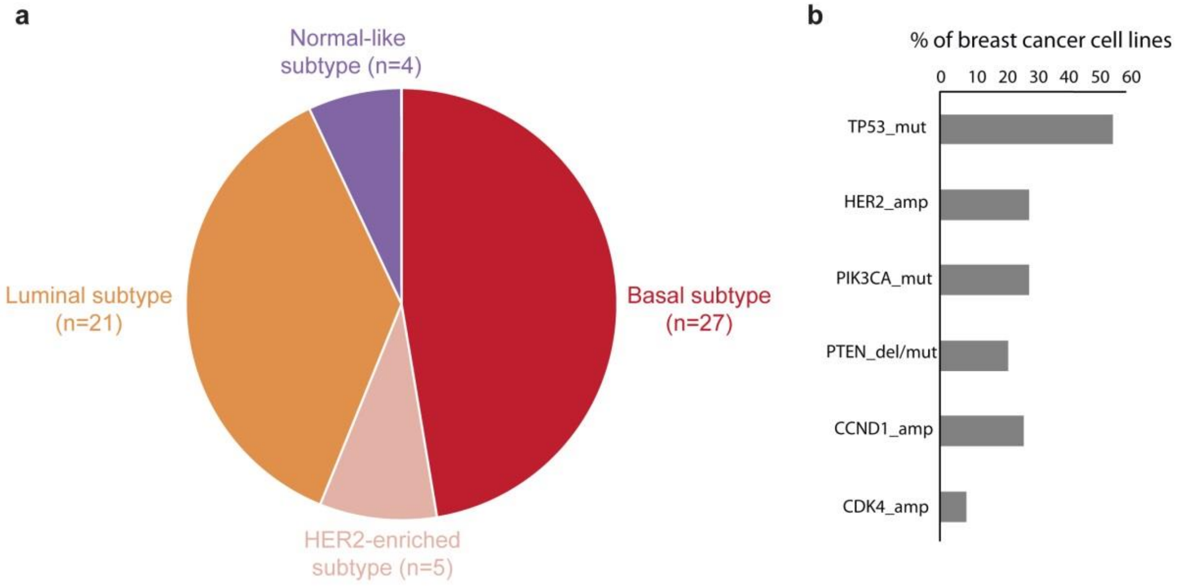

Supplementary Figure 3: Breast cancer cell lines from the cancer cell line encyclopedia represent all molecular subtypes of the human disease and exhibit the common mutations identified in the tumors.

(**a**) A pie chart describing the PAM50 molecular subtypes of the 57 breast cancer cell lines, as evaluated by expression signature analysis. (**b**) A bar chart showing the prevalence of common breast cancer mutations/alterations in the breast cancer CCLE cell lines.

Supplementary Figure 4

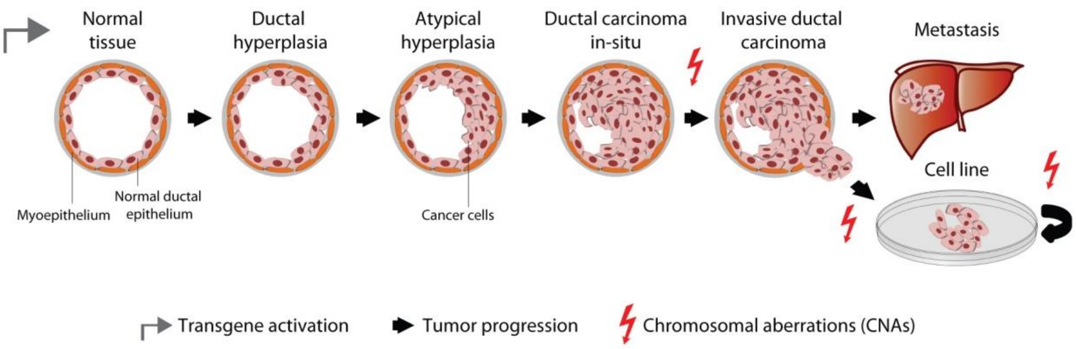

Supplementary Figure 4: Schematics of the timeline of CNA acquisition/selection during GEMM breast cancer tumorigenesis.

This schematic model describes the timeline of breast cancer tumorigenesis. The major wave of genomic instability (i.e., acquisition or selection of chromosomal aberrations) occurs during the progression of a pre-malignant tissue to an invasive carcinoma; additional waves of instability arise during cell line derivation and propagation.

Supplementary Figure 5

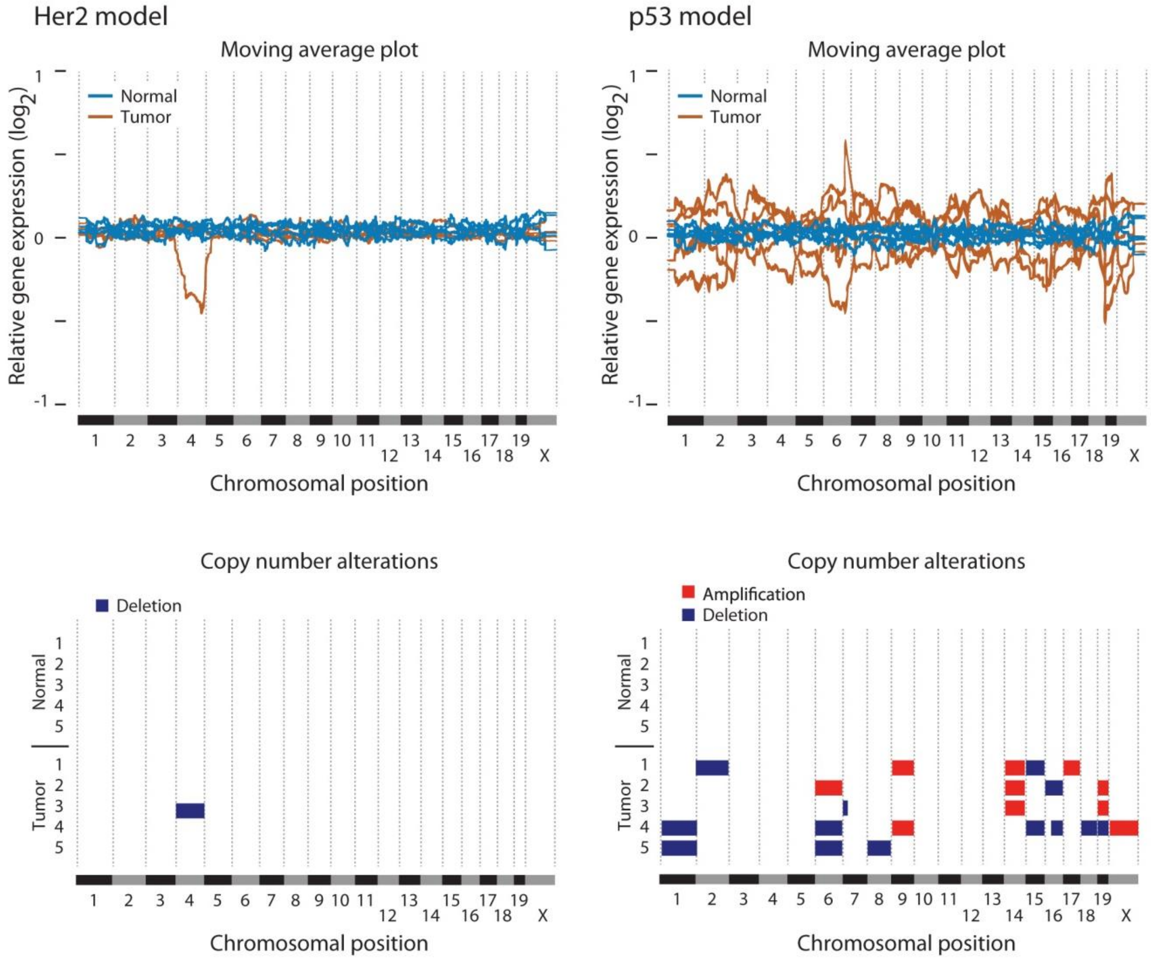

Supplementary Figure 5: Representative results from CNA analysis of genomically stable and unstable GEMMs.

Upper panel: moving average plots of global gene expression levels along the genome of 5 normal mammary samples (blue lines) and 5 tumor samples (orange lines) from two mouse models generated in the same study (GSE223938): the Her2/Neu model (left) and the $p53^{-/-}$ model (right). Lower panel: piecewise constant fit (PCF) detection of amplifications (in red) and deletions (in blue) in the same samples. Note the significant DGI difference between the two GEMMs. The data from the $p53^{-/-}$ model are the same as in **Fig. 1a**.

Supplementary Figure 6

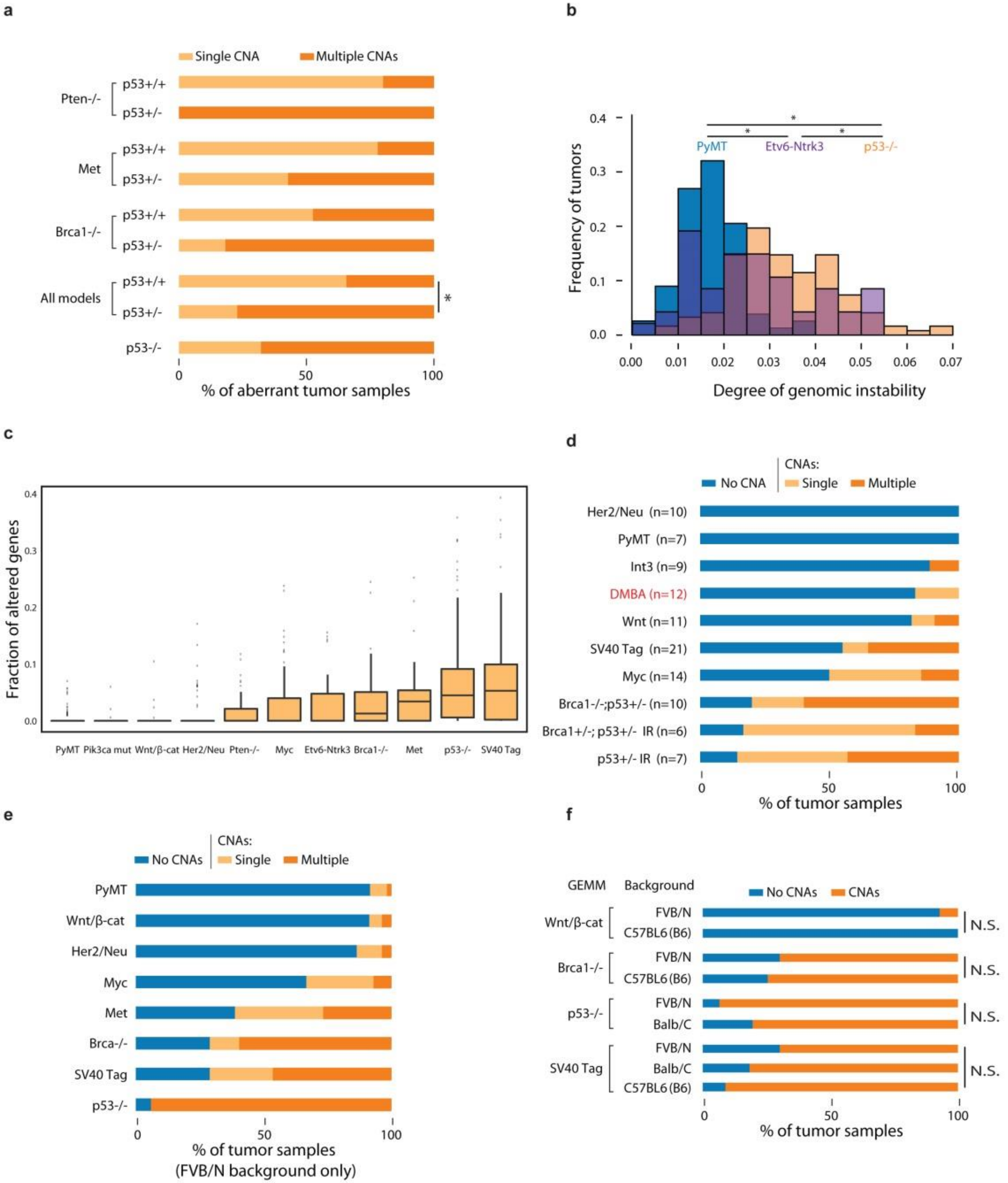

Supplementary Figure 6: Driver-specific degree of genomic instability in breast cancer GEMMs.

(**a**) The status of p53 is a major determinant of genomic instability in breast cancer GEMMs. Presented is a comparison of genomic instability in models with data available for both $p53^{+/+}$ and $p53^{+/-}$ background. *, $p=2x10^{-4}$ (Fisher's exact test). (**b**) The degree of genomic instability, as estimated by autocorrelation between proximate genes, in three representative GEMMs. *,$p=1.2x10^{-14}$, $p=5.5x10^{-3}$ and $p=2.2x10^{-16}$ (Mann-Whitney U test), for the PyMT/Etv6-Ntrk3, Etv6-Ntrk3/p53 and PyMT/p53 comparisons, respectively. (**c**) The degree of genomic instability, as estimated by the proportion of genes affected by CNAs, across the 11 most common GEMMs. (**d**) The prevalence of chromosomal aberrations in tumors from nine genetic models and one chemical model (DMBA) of breast cancer (GSE3165), showing that some genetic models are as stable as the chemical model. (**e**) The degree of genomic instability observed in the breast cancer GEMMs is not a result of the mouse strains used for their generation. Shown is the prevalence of chromosomal aberrations in 784 tumors from eight GEMMs generated on the FVB/N strain background. The observed DGI of each model is highly similar to that observed when all strains are analyzed together (compare to **Fig. 3a**). (**f**) Also shown is the prevalence of chromosomal aberrations in four GEMMs, each generated on more than one strain background. No significant differences in DGI are observed between different backgrounds within each model.

Supplementary Figure 7

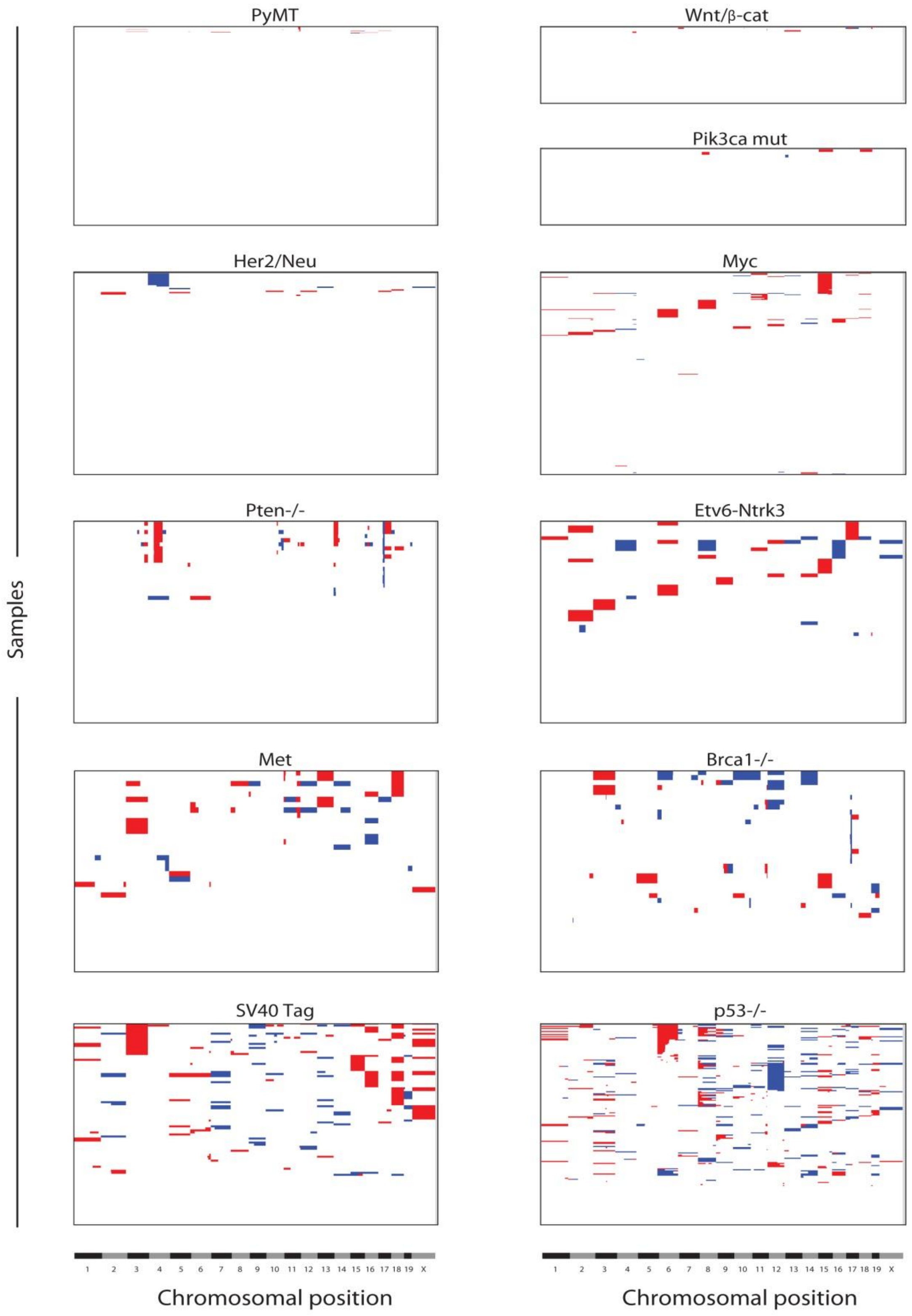

Supplementary Figure 7: Unique landscapes of aneuploidy and large CNAs in breast cancer GEMMs.

Heat maps of the chromosomal landscapes of the 11 GEMMs analyzed. Gains are shown in red, losses in blue. Heat maps correspond to the frequency plots presented in **Fig. 4a**.

Supplementary Figure 8

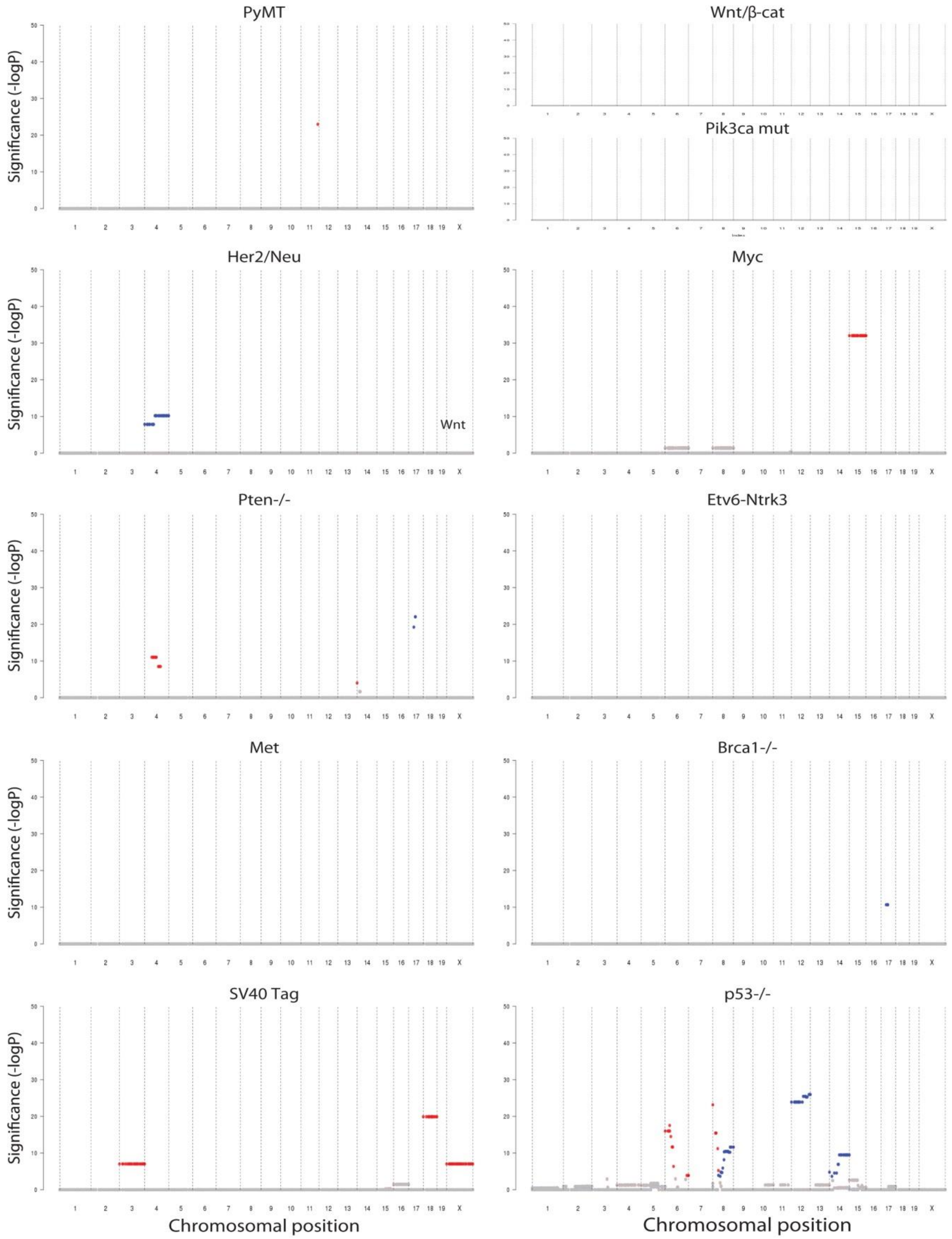

Supplementary Figure 8: Binomial distribution analysis of recurrent CNAs in breast cancer GEMMs.

Binomial distribution test for recurrence of chromosomal aberrations in each of the 11 GEMMs analyzed. Red dots denote significantly gained regions, and blue dots denote significantly lost regions (Bonferroni-corrected $p < 0.05$).

Supplementary Figure 9

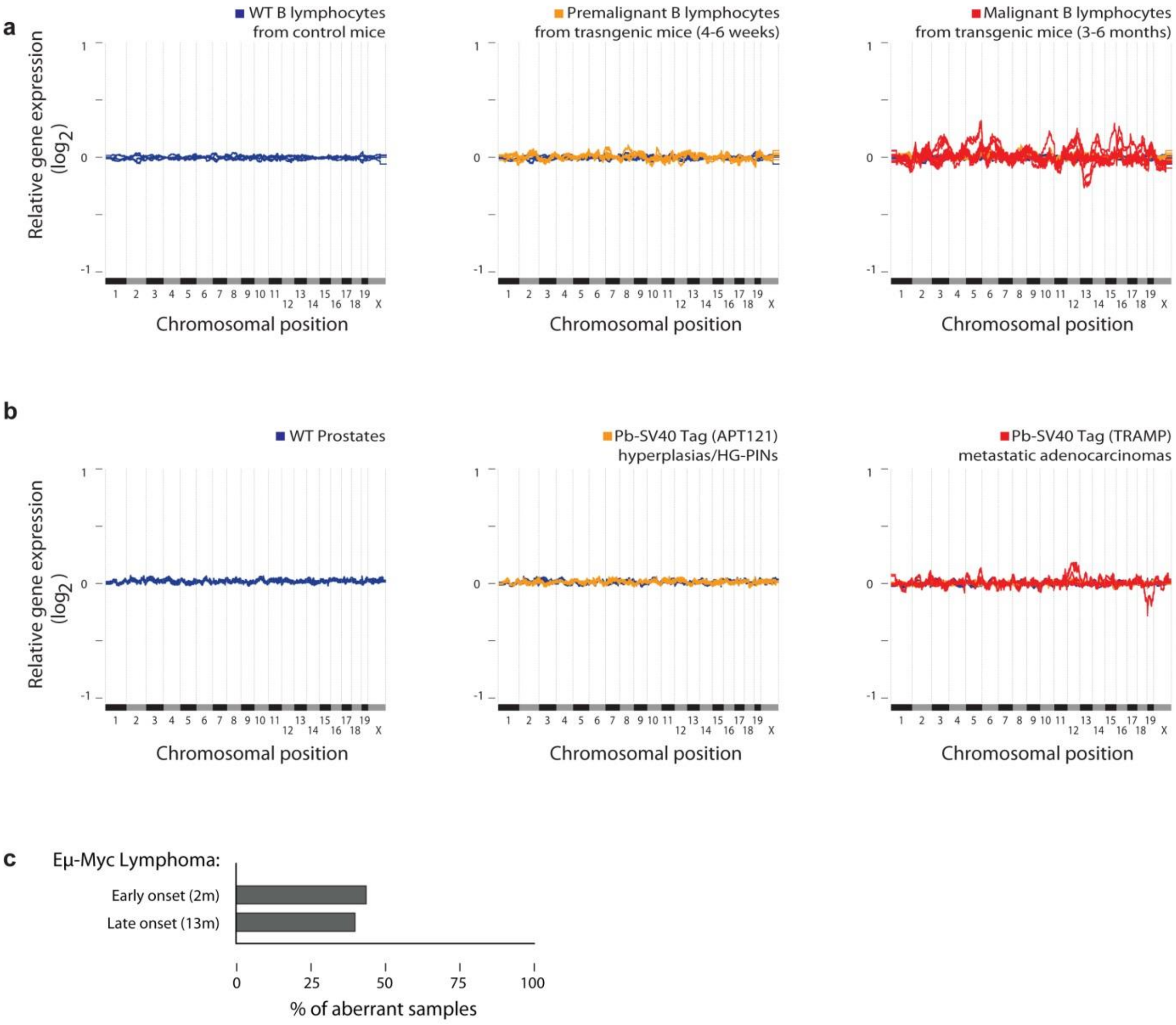

Supplementary Figure 9: Chromosomal aberrations are a late event in lymphoma and prostate cancer GEMMs.

(**a**) Following chromosomal aberrations in the Eμ-Myc mouse model of lymphoma (GSE32239). Presented are moving average plots of gene expression profiles from wildtype B lymphocytes from control mice, premalignant B lymphocytes from transgenic mice, and malignant B lymphocytes from transgenic mice. (**b**) Following chromosomal aberrations in the SV40Tag mouse models of prostate cancer (GSE53202). Presented are moving average plots of gene expression profiles from wildtype prostate tissues, hyperplasias and/or prostatic intraepithelial neoplasias (PINs) from tumors in the Pb-TagAPT$_{121}$ (APT) model, and adenocarcinomas from the Pb-T/tag (TRAMP) model. (**c**) The prevalence of chromosomal aberrations in intrinsic to the model and not determined by tumor latency in the lymphoma Eμ-Myc GEMM.

Supplementary Figure 10

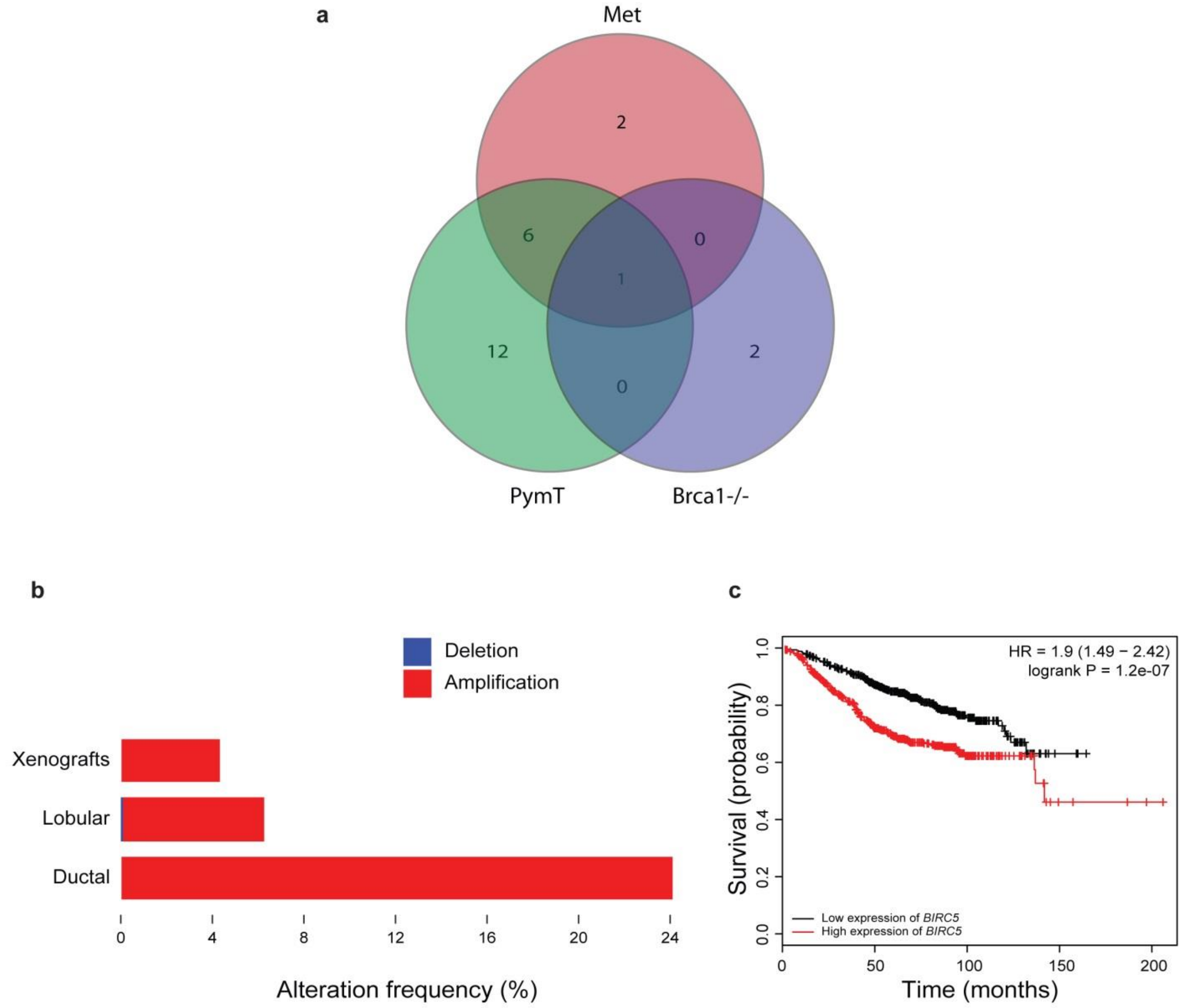

Supplementary Figure 10: Comparative oncogenomics identifies BIRC5 as a general oncogene that promotes tumorigenesis across breast cancer subtypes.

(**a**) A Venn diagram presenting the number of over-expressed genes in three GEMMs with recurrent amplification of 11qE1-E2: PyMT, Brca1$^{-/-}$ and Met. In each model, expression levels were compared between tumors and normal mammary tissues [10]. Only one gene, *Birc5*, was significantly over-expressed in all three models. (**b**) Alteration frequency of BIRC5 in three large-scale cancer genomic studies, revealing that BIRC5 is commonly amplified in human breast cancer. Data were obtained from cBioPortal [53] (http://www.cbioportal.org/). (**c**) High expression of BIRC5 is associated with worse prognosis in human breast cancer patients. Presented are Kaplan-Meier plots of patients' overall survival, based on a cohort of 1,117 patients of all molecular subtypes [46].

Supplementary Figure 11

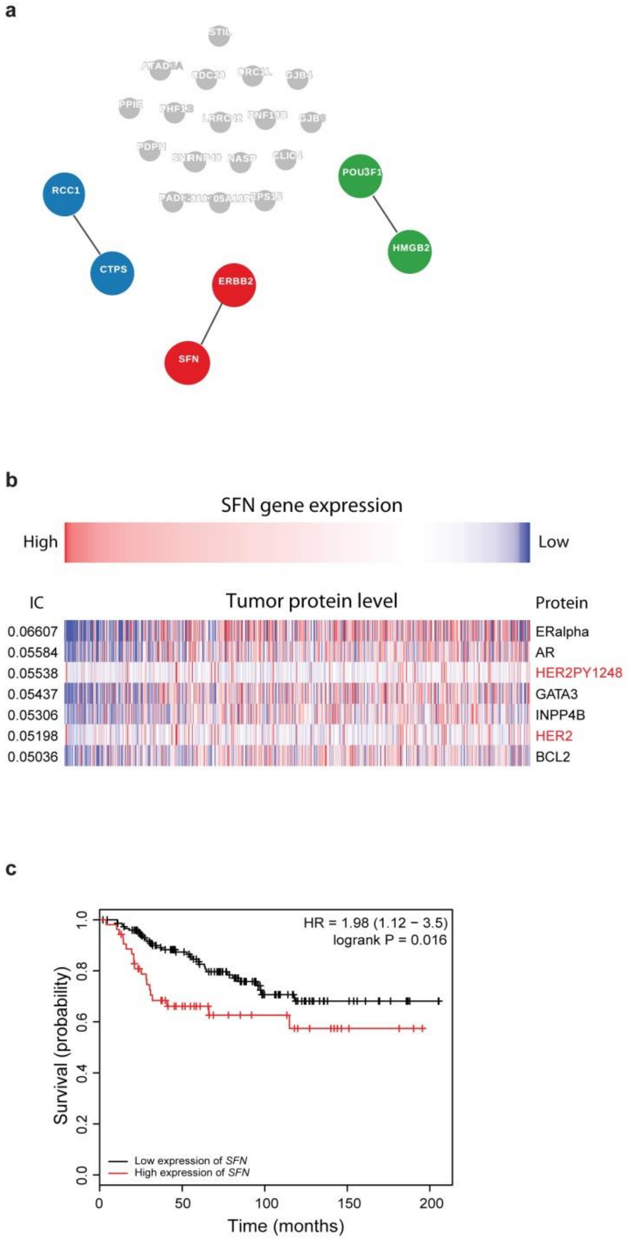

Supplementary Figure 11: Comparative oncogenomics identifies SFN as a putative co-driver gene that cooperates with HER2 during breast tumorigenesis.

(**a**) Out of the 22 genes that reside within mouse chromosome 4 and the syntenic region on human chromosome 1p, and are downregulated in the Her2/Neu GEMM, only two genes were found to be connected to *HER2* in a network analysis: *SFN* and *EPS15*. Of these, only SFN interacts directly with HER2 in a protein-protein interaction (PPI) analysis, shown here. (For reference, a PPI analysis of HER2 together with all human 1p genes identifies ~40 times as many interactions.) (**b**) *SFN* gene expression level is anti-correlated with the protein expression level of HER2 in human breast tumors. (**c**) High expression of *SFN* is associated with worse prognosis in basal subtype tumors, in line with previous findings [35]. Presented are Kaplan-Meier plots of patients' overall survival, based on a cohort of 204 basal subtype patients [46].

Supplementary Figure 12

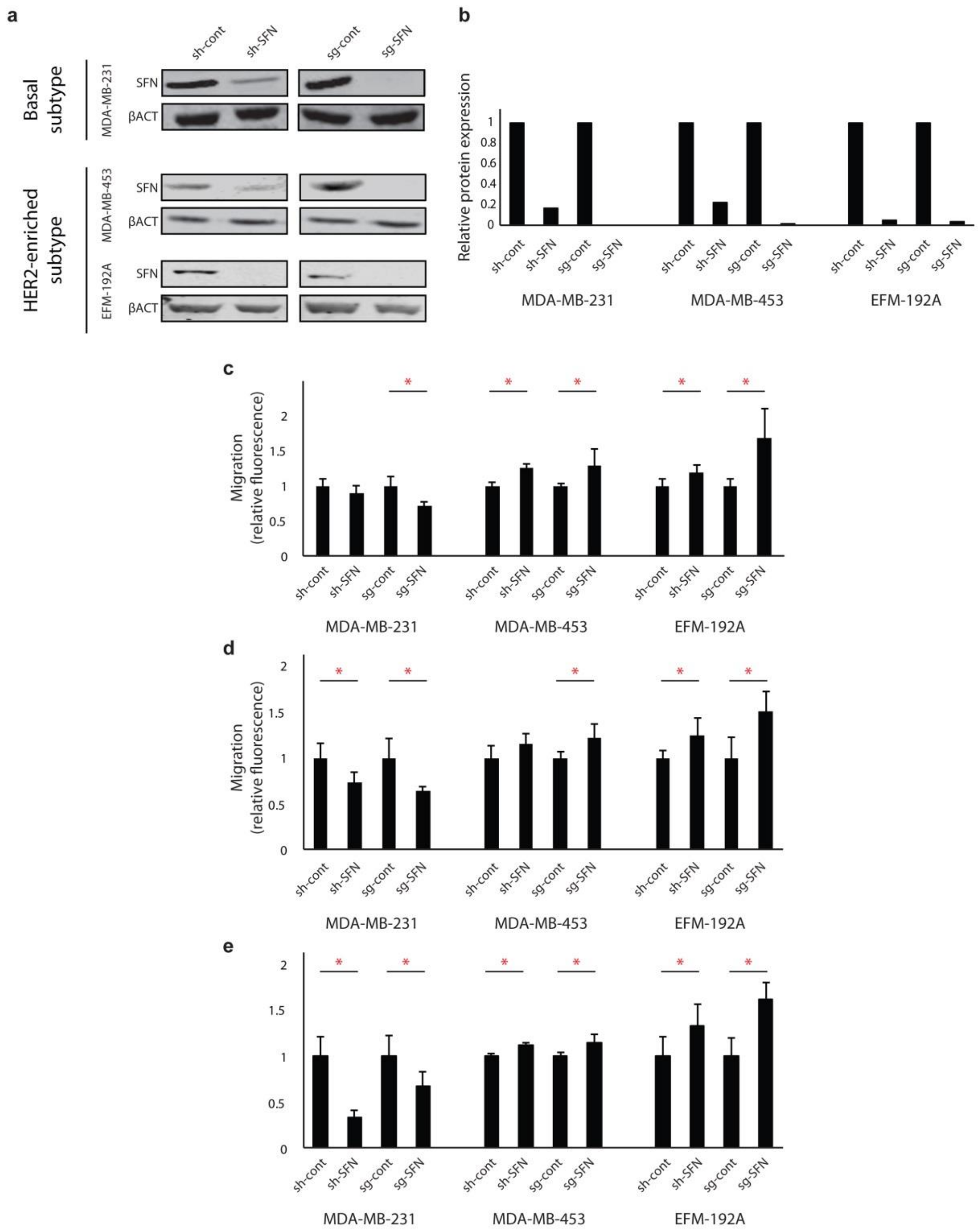

Supplementary Figure 12: Downregulation of SFN promotes *in vitro* tumorigenesis of human breast cancer cell lines of the *HER2*-enriched subtype.

(a) Immunoblot analysis of SFN protein levels in human breast cancer cell lines, following shRNA-mediated knockdown or CRISPR/Cas9-mediated knockout of SFN. **(b)** A quantification of the reduction in protein levels. **(c)** Decreased migration of the basal cell line and increased migration of the *HER2*-enriched cell lines, following the knockdown/knockout of SFN, as evaluated by a transwell migration assay. *, $p<0.05$ (Student's t-test). **(d)**. Decreased invasion of the basal cell line and increased invasion of the *HER2*-enriched cell lines, following the knockdown/knockout of SFN, as evaluated by a transwell invasion assay. *, $p<0.05$ (Student's t-test). (**e**) Decreased colony formation of the basal cell line and increased colony formation of the *HER2*-enriched cell lines, following the knockdown/knockout of SFN, as evaluated by a soft-agar assay. *, $p<0.05$ (Student's t-test).

Supplementary Figure 13

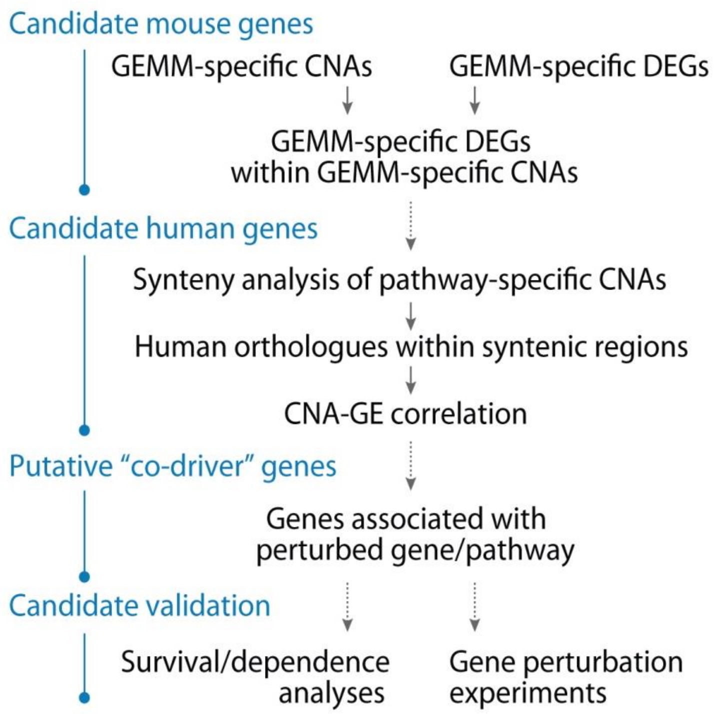

Supplementary Figure 13: Comparative oncogenomics pipeline to identify candidate co-driver genes underlying the recurrence of driver-specific CNAs.

Schematic outlining of the strategy that we applied to the driver-specific CNAs. The candidate genes identified by this strategy are listed in **Supplementary Data 9**. GEMMs, genetically-engineered mouse models; CNAs, copy number alterations; DEGs, differentially expressed genes; GE, gene expression.

Supplementary Figure 14

Figure 5c

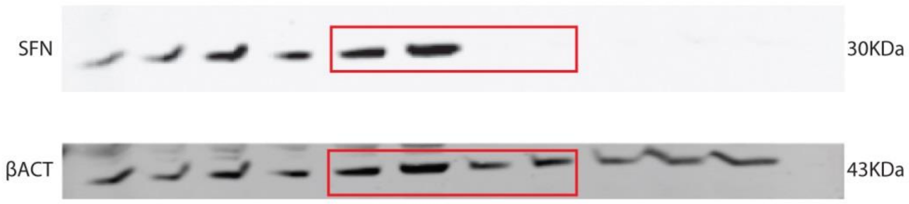

Figure 5d

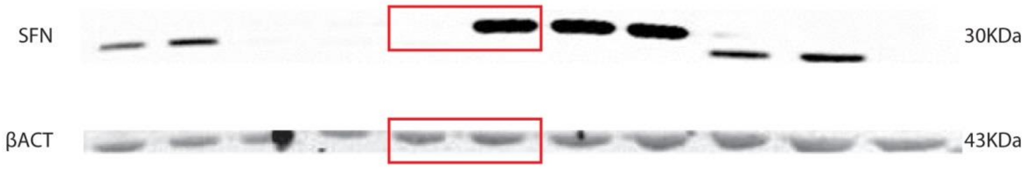

Supplementary Figure 14: Uncropped western blots

Uncropped scans of western blots displayed in the main Figures

Supplementary Table 1

Kruskal-Wallis rank sun test by altered genes:
Chi-squared=595.2952, df=10, p<0.0001

	BRCA1-/-	Etv6-Ntrk3	Her2/Neu	Met	Myc	Pik3ca	Pten-/-	PyMT	SV40 Tag	Wnt/β-cat
Etv6-Ntrk3	1									
Her2/Neu	<0.0001	<0.0001								
Met	1	1	<0.0001							
Myc	0.0025	1	<0.0001	0.0102						
Pik3ca	<0.0001	0.0107	1	0.0001	0.1127					
Pten-/-	0.7299	1	0.0075	0.9746	1	0.1368				
PyMT	<0.0001	<0.0001	1	<0.0001	<0.0001	1	0.0001			
SV40 Tag	0.6286	0.0003	<0.0001	0.9358	<0.0001	<0.0001	0.0001	<0.0001		
Wnt/β-cat	<0.0001	0.0005	1	<0.0001	0.0035	1	0.0229	1	<0.0001	
p53-/-	0.3438	<0.0001	<0.0001	0.5995	<0.0001	<0.0001	<0.0001	<0.0001	1	<0.0001

Kruskal-Wallis rank sun test by CNA prevalence (# of events per sample):
Chi-squared=739.3707, df=10, p<0.0001

	BRCA1-/-	Etv6-Ntrk3	Her2/Neu	Met	Myc	Pik3ca	Pten-/-	PyMT	SV40 Tag	Wnt/β-cat
Etv6-Ntrk3	0.3156									
Her2/Neu	<0.0001	<0.0001								
Met	1	1	<0.0001							
Myc	<0.0001	0.0066	0.0004	0.0021						
Pik3ca	<0.0001	0.0011	1	0.0003	0.9074					
Pten-/-	0.0022	1	0.0018	1	1	0.1686				
PyMT	<0.0001	<0.0001	1	<0.0001	<0.0001	1	<0.0001			
SV40 Tag	1	0.0482	<0.0001	1	<0.0001	<0.0001	<0.0001	<0.0001		
Wnt/β-cat	<0.0001	<0.0001	1	<0.0001	0.0199	1	0.0063	1	<0.0001	
p53-/-	1	0.0002	<0.0001	0.0553	<0.0001	<0.0001	<0.0001	<0.0001	1	<0.0001

Supplementary Table 1: Statistically significant DGI differences between breast cancer GEMMs

The variation analysis between the 11 most common breast cancer GEMMs was performed by a one-way ANOVA on ranks (Kruskal-Wallis) test, followed by a post-hoc Dunn's test to compare each pair of GEMMs. Shown are the Bonferroni corrected p-values for the pair-wise analyses, based either on CNA prevalence (i.e., number of aberrations per sample) or on the proportion of altered genes per sample. Pair-wise comparisons revealed statistically-significant differences between low, medium and high DGI models.

Supplementary Table 2

Mouse model	Recurrent aberrations (>10% of sampels, regardless of statistical significance)	Recurrent aberrations (Statistically significant by a binomial distribution test)	Frequency of aberrations (regional minimum/maximum)	Binomial test p-value for significance within model (maximum of region)	GISTIC2.0 q-value for significance within model (maximum of region)	Chi-squared test p-value for significance between models (maximum of region)
**PymT**	-	Amp 11qE1-E2	0.022/0.036	1.60E-10	3.15E-44	N.S.
**Wnt/β-cat**	-	-	-	-	-	-
**Pik3ca**	-	-	-	-	-	-
**Her2/Neu**	-	Del 4	0.060/0.067	3.90E-04	0.00E+00	2.00E-03
**Myc**	Amp 15	Amp 15	0.102	1.20E-14	0.00E+00	4.20E-04
**Pten-/-**	Amp 4qB1-C6	Amp 4qB1-C6	0.184/0.204	2.00E-04	1.65E-09	2.00E-41
	Amp 14qA1-A3	Amp 14qA1	0.122/0.142	1.80E-02	2.52E-05	2.60E-11
	Del 17qB1-B3	Del 17qB1-B3	0.286/0.306	4.40E-09	1.32E-14	7.42E-36
	Amp 3qG3-H4	-	0.102	N.S.	2.60E-03	N.S.
	Amp 17qC-E5	-	0.102	N.S.	2.60E-03	N.S.
**Etv6-Ntrk3**	Amp 2	-	0.111	N.S.	1.50E-03	1.20E-05
**Met**	Amp 3	-	0.105/0.132	N.S.	6.62E-03	N.S.
	Amp 11qA1	-	0.105	N.S.	N.S.	6.80E-04
	Amp 11qE1-E2	-	0.132	N.S.	1.30E-02	N.S.
	Amp 13	-	0.105	N.S.	2.28E-02	4.68E-17
	Amp 18	-	0.132	N.S.	6.62E-03	N.S.
**BRCA1-/-**	Amp 3qF2.1	Amp 3qF2.1	0.122	N.S.	2.57E-02	N.S.
	Amp 11qE2	Amp 11qE2	0.146	N.S.	2.58E-04	N.S.
	Del 17qB1-B3	Del 17qB1-B3	0.317	2.30E-05	4.53E-18	4.60E-35
	Del 6qA1-A3.2	-	0.122	N.S.	2.34E-04	2.20E-16
	Del 10qD2-D3	-	0.122	N.S.	5.77E-04	9.60E-07
	Del 12qA1.1-D2	-	0.122	N.S.	2.56E-03	N.S.
**SV40 Tag**	Amp 3	Amp 3	0.174	8.80E-04	1.53E-09	1.10E-09
	Amp 18	Amp 18	0.25	2.30E-09	1.11E-14	2.70E-38
	Amp X	Amp X	0.174	8.80E-04	1.53E-09	9.40E-45
	Amp 15	-	0.109	N.S.	1.23E-02	N.S.
	Amp 16	-	0.13	N.S.	1.72E-03	1.15E-10
	Del 9qA5.1-A5.3	-	0.108	N.S.	3.11E-03	1.20E-16
**p53-/-**	Amp 6qA1-C1	Amp 6qA1-B3	0.121/0.163	1.70E-03	1.30E-05	4.10E-12
	Amp 6qG1-G3	Amp 6qG1-G3	0.111	2.00E-02	1.70E-02	1.45E-06
	Amp 8qA1.1-A4	Amp 8qA1.1-A4	0.111/0.179	5.30E-03	2.08E-14	3.30E-19
	Del 8qB1.2-E2	Del 8qB1.1-E2	0.105/0.137	2.50E-02	1.60E-10	1.00E-28
	Del 12	Del 12	0.179/0.184	4.20E-11	0.00E+00	3.40E-43
	Del 14	Del 14	0.105/0.132	1.00E-02	2.02E-08	2.10E-18
	Amp 3qF2.1	-	0.105	N.S.	2.11E-02	4.40E-05
	Amp 15qA1-C	-	0.105	N.S.	3.60E-02	N.S.

Supplementary Table 2: Recurrent CNAs in breast cancer mouse models

A summary of the recurrent aberrations in the 11 most common breast cancer GEMMs. Presented are all aberrations present in>10% of tumor samples, and those identified as significant by a binomial distribution test. 34 of the 35 events were confirmed to be significant by a GISTIC2.0 analysis. Recurrent aberrations were subjected to a chi-squared test, to examine their model-specificity. Adjusted p-values are mentioned for the binomial and chi-squared tests, and q-values are mentioned for the GISTIC2.0 analysis. Significant model-specific recurrent events are highlighted in blue.

Supplementary Table 3

Mouse Model	Recurrent transgene-specific aberrations (statistically significant in both tests)	Human chromosomes containing synteny blocks	Human synteny blocks	Syntenic regions significantly altered in the same direction in human breast cancer with the activation of the same pathway
**Her2/Neu**	Del 4	1,6,8,9	Chr 1:0.9M-61.7M, Chr 6:87.1M-99.8M, Chr 8:55.7M-61.8M, Chr 8:86.0M-96.2M, Chr 9:6.8M-38.5M, Chr 9:80.4M-83.6M, Chr 9:97.3M-120.7M	Chr 1p: 0.9M-61.7M
**Myc**	Amp 15	5,8,12,22	Chr 5:8.9M-42.9M, Chr 8:96.4M-144.6M, Chr 12:33.1M-34.1M, Chr 12:38.2M-54.7M, Chr 22:35.6M-50.8M	Chr 8q: 96.4M-144.6M
**p53-/-**	Amp 6qA1-B1	7	Chr 7:93.1M-97.9M, Chr 7:7.1M-12.5M, Chr 7:112.5M-128.5M, Chr 7:128.7M-149.9M	-
	Amp 6qG1-G3	12	Chr 12:9.7M-32.4M	Chr 12p: 9.7M-32.4M
	Amp 8qA1.1-A4	8,13, 19	Chr 8:0.4M-18.1M, Chr 13:102.9M-114.3M, Chr 19:7.1M-8.1M	-
	Del 8qB1.1-E2	1,4,8,10,16,19,21,22	Chr 1:229.2M-235.2M, Chr 4:140.3M-150.0M, Chr 4:162.6M-190.0M, Chr 8:18.1M-20.3M, Chr 10:32.8M-34.9M, Chr 19:12.6M-14.6M, Chr 19:16.1M-19.7M, Chr 22:33.3M-35.5M, Chr 16:46.7-90.0M	Chr 4q:140.3M-150.0M, Chr 4q:162.6M-190.0M, Chr 8:18.1M-20.3M
	Del 12	2,7,9,14	Chr 12:0.2M-17.8M, Chr 2:94.6M-94.7M, Chr 7:12.5M-22.5M, Chr 7:105.6M-112.5M, Chr 7:157.4M-159.1M, Chr 9:40.4M-40.5M, Chr 9:43.0M-43.1M, Chr 9:64.5M-66.0M, Chr 14:24.7M-51.8M, Chr 14:58.2M-105.9M	Chr 14q:24.7M-51.8M, Chr 14q:58.2M-105.9M
	Del 14	3,6,8,10,13,14	Chr 3:15.2M-16.3M, Chr 3:23.1M-27.7M, Chr 3:52.3M-64.0M, Chr 6:39.1M-39.3M, Chr 8:9.8M-11.9M, Chr 8:20.3M-29.3M, Chr 10:45.9M-50.0M, Chr 10:73.1M-79.5M, Chr 10:80.3M-87.2M, Chr 13:19.6M-24.9M,	Chr 8p:9.8M-11.9M, Chr 8p:20.3M-29.3M, Chr 14q:19.7M-24.7M, Chr 14q:52.2M-58.2M,

			Chr 13:40.9M-102.4M, Chr 14:19.7M-24.7M, Chr 14:52.2M-58.2M,	
**Pten-/-**	Amp 4qB1-C6	1,9	Chr 1:58.7M-67.1M, Chr 9:6.8M-38.5M, Chr 9:80.4M-83.6M, Chr 9:97.3M-120.7M	-
	Amp 14qA1	3	Chr 3:58.0M-64.0M	-
	Del 17qB1-B3	6,19,21	Chr 6:29.4M-33.3M, Chr 19:8.3M-8.7M, Chr 19:15.2M-15.7M, Chr 21:42.1M-43.7M	-
**Brca1-/-**	Del 17qB1-B3	6,19,21	Chr 6:29.4M-33.3M, Chr 19:8.3M-8.7M, Chr 19:15.2M-15.7M, Chr 21:42.1M-43.7M	-
**SV40 Tag**	Amp 3	NA	NA	-
	Amp 18	NA	NA	-
	Amp X	NA	NA	-

Supplementary Table 3: Syntenic aberrations in breast cancer GEMMs and in human breast tumors that activate the same pathway

A summary of the 15 model-specific CNAs identified in breast cancer GEMMs, together with their syntenic human chromosomal regions. Highlighted are syntenic regions that are significantly altered in the same direction in human tumors that activate the same pathway, as judged by gene expression signatures [33].
